# CD73^+^ CD127^high^ Long-Term Memory CD4 T Cells Are Highly Proliferative in Response to Recall Antigens and Are Early Targets in HIV-1 Infection

**DOI:** 10.3390/ijms22020912

**Published:** 2021-01-18

**Authors:** Nabila Seddiki, John Zaunders, Chan Phetsouphanh, Vedran Brezar, Yin Xu, Helen M. McGuire, Michelle Bailey, Kristin McBride, Will Hey-Cunningham, Cynthia Mee Ling Munier, Laura Cook, Stephen Kent, Andrew Lloyd, Barbara Cameron, Barbara Fazekas de St Groth, Kersten Koelsch, Mark Danta, Hakim Hocini, Yves Levy, Anthony D. Kelleher

**Affiliations:** 1 INSERM U955, Equipe 16, 94000 Créteil, France; vedranbrezar@gmail.com (V.B.); hakim.hocini@inserm.fr (H.H.); yves.levy@hmn.aphp.fr (Y.L.); 2 Faculté de médecine, Université Paris-Est Créteil, 94000 Créteil, France; 3 Vaccine Research Institute (VRI), 94000 Créteil, France; 4 Centre for Applied Medical Research, St Vincent’s Hospital, Sydney, NSW 2010, Australia; 5 Kirby Institute, University of New South Wales, Sydney, NSW 2052, Australia; cphetsouphanh@kirby.unsw.edu.au (C.P.); unsw3086075@gmail.com (Y.X.); Michelle.Bailey@awe.gov.au (M.B.); k.mcbride@unsw.edu.au (K.M.); willheyco@gmail.com (W.H.-C.); cmunier@kirby.unsw.edu.au (C.M.L.M.); lcook@bcchr.ca (L.C.); a.lloyd@unsw.edu.au (A.L.); kkoelsch@me.com (K.K.); akelleher@kirby.unsw.edu.au (A.D.K.); 6 Charles Perkins Centre, The University of Sydney, Sydney, NSW 2006, Australia; helen.mcguire@sydneycytometry.org.au (H.M.M.); barbara.fazekas@sydney.edu.au (B.F.d.S.G.); 7 Ramaciotti Facility for Human Systems Biology, The University of Sydney, Sydney, NSW 2006, Australia; 8 Discipline of Pathology, Faculty of Medicine and Health, The University of Sydney, Sydney, NSW 2006, Australia; 9 Department of Microbiology and Immunology, University of Melbourne, Melbourne, VIC 3010, Australia; skent@unimelb.edu.au; 10 School of Medical Sciences, University of New South Wales, Sydney, NSW 2052, Australia; b.cameron@unsw.edu.au; 11 St Vincent’s Clinical School, St Vincent’s Hospital and University of New South Wales, Sydney, NSW 2052, Australia; m.danta@unsw.edu.au; 12 AP-HP, Hôpital H. Mondor—A. Chenevier, Service d’immunologie Clinique et Maladies Infectieuses, 94000 Créteil, France

**Keywords:** CD73, CD4+ subsets, HIV-1 viral reservoir

## Abstract

HIV-1 infection rapidly leads to a loss of the proliferative response of memory CD4+ T lymphocytes, when cultured with recall antigens. We report here that CD73 expression defines a subset of resting memory CD4+ T cells in peripheral blood, which highly express the α-chain of the IL-7 receptor (CD127), but not CD38 or Ki-67, yet are highly proliferative in response to mitogen and recall antigens, and to IL-7, in vitro. These cells also preferentially express CCR5 and produce IL-2. We reasoned that CD73+ memory CD4+ T cells decrease very early in HIV-1 infection. Indeed, CD73+ memory CD4+ T cells comprised a median of 7.5% (interquartile range: 4.5–10.4%) of CD4+ T cells in peripheral blood from healthy adults, but were decreased in primary HIV-1 infection to a median of 3.7% (IQR: 2.6–6.4%; *p* = 0.002); and in chronic HIV-1 infection to 1.9% (IQR: 1.1–3%; *p* < 0.0001), and were not restored by antiretroviral therapy. Moreover, we found that a significant proportion of CD73+ memory CD4+ T cells were skewed to a gut-homing phenotype, expressing integrins α4 and β7, CXCR3, CCR6, CD161 and CD26. Accordingly, 20% of CD4+ T cells present in gut biopsies were CD73+. In HIV+ subjects, purified CD73+ resting memory CD4+ T cells in PBMC were infected with HIV-1 DNA, determined by real-time PCR, to the same level as for purified CD73-negative CD4+ T cells, both in untreated and treated subjects. Therefore, the proliferative CD73+ subset of memory CD4+ T cells is disproportionately reduced in HIV-1 infection, but, unexpectedly, their IL-7 dependent long-term resting phenotype suggests that residual infected cells in this subset may contribute significantly to the very long-lived HIV proviral DNA reservoir in treated subjects.

## 1. Introduction

CD73 belongs to the family of ectonucleotidases that hydrolyze extracellular nucleotides and are widely expressed multifunctional cell surface molecules, affecting the cellular milieu, providing products for cell signaling, and in some cases mediating cellular adhesion and migration [1]. Murine CD4+ T regulatory cells (Tregs) express two ecto-nucleotidases, the ATPase CD39 and the ecto-5′-nucleotidase CD73 [2], such that their combined activity can sequentially catabolize ATP to ADP and AMP, and then AMP to adenosine, respectively, and thereby suppress lymphocyte activation [2]. However, in the case of circulating human Tregs, using the CD25^high^CD127^low^FOXP3+ phenotype [3], it has been shown that a subset of human Tregs express CD39, and do not express CD73 [4,5,6,7]. The possibility remains that close proximity of CD39+ Tregs and CD73+ cells acting together may generate adenosine [8].

It had previously been reported that ecto-5′-nucleotidase activity was reduced in PBMC in subjects with HIV-1 infection [9], and more recent studies have shown that human memory CD73+ subset of CD4+ T cells, distinct from Tregs, was reduced in percentage, and also in absolute number, in peripheral blood samples from HIV-infected subjects [6,10]. Similarly, decreases in CD73+ CD8+ T cells [6] and CD73+ B cells [11] have also been reported in HIV+ subjects. Therefore, the loss of many CD73+ lymphocytes and a consequent reduction in extracellular adenosine concentration has been proposed to lead to loss of homeostatic suppression and contribute to the elevation of lymphocyte activation that is characteristic of HIV-1 infection [6,11,12].

Elevated activation of T lymphocytes is evident from the earliest stages of primary HIV-1 infection (PHI) [13] and peaks prior to the transition to chronic infection [14]. Therefore, we have studied the CD73+ subset of memory CD4+ T cells in during PHI, their susceptibility to HIV infection in vivo, and the effect of commencing antiretroviral therapy (ART) at that stage of infection, versus late in infection.

Additionally, CD73 expression on lymphocytes may be involved in specialized cell migration [1], resulting in trafficking patterns for CD73+ cells that differ from other lymphocytes. In murine studies, CD73-deficient T cells failed to enter lymph nodes via an afferent lymph, compared with wild-type T cells [15], and also exhibited increased extravasation across high endothelial venules (HEV), compared with CD73 wild-type cells that were blocked at the HEV [16]. Taken together, this suggests that CD73+ lymphocytes follow a dedicated recirculation pattern via afferent lymphatic vessels into peripheral lymph nodes, rather than following naïve and central memory T cells, which extravasate across HEV [17,18]. In another study, CD73-deficient T cells did not enter the central nervous system (CNS) during experimental autoimmune encephalitis [19], again suggesting an important role for CD73 in specialized extravasation of lymphocytes.

Therefore we have studied in detail, the expression of trafficking markers on CD73+ CD4+ T cells, since their anatomical location, relative to other lymphocyte subsets, will determine their role in regulating immune responses.

Similarly, the antigen specificity and function of CD73+ CD4+ T cells will be important in understanding the effect of their loss on the pathogenesis of HIV-1 infection. We therefore studied recall responses of CD73+ CD4+ T cells for a range of common memory CD4+ T cell antigen specificities, such as CMV, tetanus toxoid, HSV, vaccinia virus and *M. tuberculosis* (Mtb), including proliferative responses and cytokine production in vitro. Of note, anti-CD73 monoclonal antibodies exhibit costimulatory activity, possibly greater than the well described costimulation via CD28, for proliferation of T cells in vitro, particularly with submitogenic concentrations of anti-CD3 [20]. Possible ligands for CD73 include extracellular matrix molecules [20] and intracellular signaling may depend on the Src family tyrosine kinase Fyn [21]. Therefore, expression of CD73 may in turn lead to enhanced proliferation and cytokine production, relative to other subsets of CD4+ T cells.

Our results demonstrate that CD73+ CD4+ T cells exhibit attributes of both effector memory and central memory cells [17] and are resting cells with high expression of the IL-7R alpha chain (CD127) and very low turnover in vivo, and yet proliferate at a high rate when incubated in vitro with well described CD4+ T lymphocyte recall antigens. Examination of trafficking markers on these cells shows that they are likely to follow a distinct recirculation pattern in vivo distinct from classical CD62L^+^CCR7^high^ central memory cells and Tregs. One of those markers, CCR5, appears to make these cells particularly vulnerable to HIV infection and our results detail not only their depletion, beginning very early in the course of primary HIV-1 infection, but also proviral infection of the remaining CD73+ CD4+ T cells.

## 2. Results

### 2.1. CD73+ Memory CD4+ T Cells in Peripheral Blood, Lymph Nodes and Cerebrospinal Fluid (CSF)

**Figure 1:**
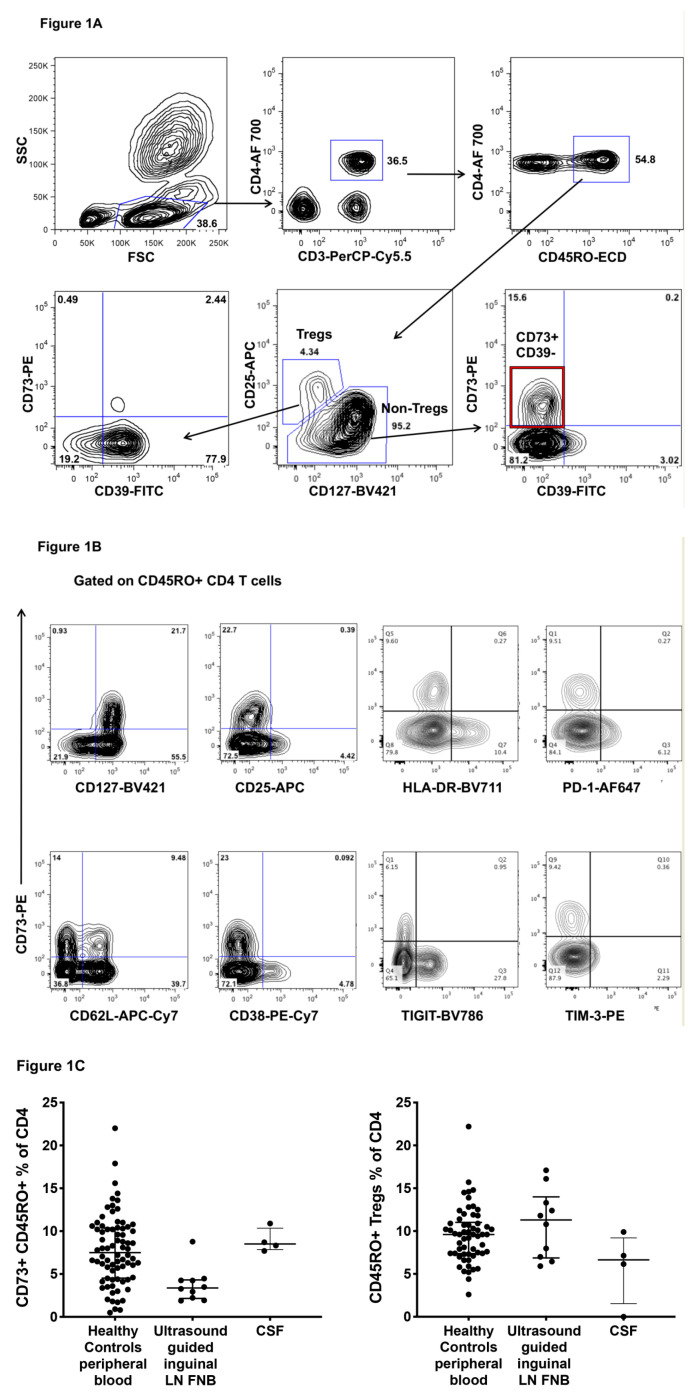
CD73+ CD4+ T cells. (**A**) Representative flowplots showing expression of CD73 on CD45RO+ memory CD4+ CD3+ lymphocytes, on the non-Treg subset (bottom right flow plot) when Tregs are gated as CD25^high^CD127^dim^. Conversely, there is minimal expression of CD73 on Tregs, which are predominantly CD39+CD73 (bottom left flowplot). (**B**) Representative flowplots showing that CD73+ CD45RO+ memory CD4+ CD3+ lymphocytes are almost exclusively CD127^high^, CD25^low^, CD38^low^, HLA-DR^low^, TIGIT^low^, PD-1^low^ and TIM-3^low^, but a mixture of CD62L+ and CD62L-negative cells. (**C**) Summary data for CD73+ cells as a percentage of CD45RO+ memory CD4+ CD3+ T lymphocytes from blood samples from *n* = 76 healthy adult controls, from ultrasound guided fine needle biopsies of inginual lymph nodes and from cerebrospinal fluid (CSF) samples from HIV+ subjects on antiretroviral therapy (ART).

We first confirmed that the CD73+ subset of human CD4+ T cells is found within the CD45RO+ memory subset of CD4+ T cells, and specifically can be detected in the CD25lowCD127high non-Treg subset of memory CD4+ T cells, but not within the CD25+CD127dim Tregs [3] (Figure 1A, bottom right flowplot). Only a very small proportion of human CD25+CD127dim Tregs coexpress CD39 and CD73 (Figure 1A, bottom left flowplot), and furthermore, not all human Tregs expressed CD39, with a median CD39+ subset of 54% of Tregs (interquartile range: 30–79%), as reported by others [4,22].

CD73+ memory CD4+ T cells were CD127high, and as such, were distinct from activated CD38+, HLA-DR+ or CD25+ memory CD4 T cells, and also were distinct from CD4 T cells expressing checkpoint inhibitors, including TIGIT, PD-1 and TIM-3 but were heterogeneous in expression of the lymph node homing marker, CD62L (Figure 1B), consistent with a mixture of central and effector memory cell phenotypes [17].

Overall, from 76 healthy adult controls we found that CD73+CD45RO+ cells as a percent of peripheral blood CD4 T cells were quite variable, with a median of 7.5% (interquartile range: 4.5–10.5%; Figure 1C).

Since murine studies suggest that CD73 is involved in trafficking to lymph nodes [15,16], we studied whether CD73+ CD4+ T cells were found in normal lymph nodes, using ultrasound-guided lymph node fine needle biopsies of inguinal lymph nodes in healthy controls [23]. The results show that a median 3.4% of CD4+ T cells in lymph nodes were CD73+ CD45RO+ (Figure 1C), which is about half the proportion of the corresponding cells in the circulation, consistent with approximately 50% of circulating CD73+ CD45RO+ cells expressing CD62L (Figure 1B, and see below). In contrast, Tregs tended to a higher proportion in memory CD4+ T cells in lymph nodes compared to peripheral blood (Figure 1C), consistent with their very high expression of CD62L (see below).

Murine CD73 knockout studies also suggest that CD73 may be involved in trafficking of T cells to the CNS [19]. We quantified the CD73+ subset of CD4+ T cells in 4 available cerebrospinal fluid (CSF) samples from HIV+ patients, on fully suppressive antiretroviral therapy being investigated for neurocognitive decline (Zaunders et al., manuscript in preparation). Proportional levels of CD73+ CD4+ T cells in CSF were comparable to peripheral blood in healthy adults (Figure 1C), whereas Tregs tended to lower proportions in CSF.

### 2.2. Detailed Phenotype of CD73+ Memory CD4+ T Cells

**Figure 2:**
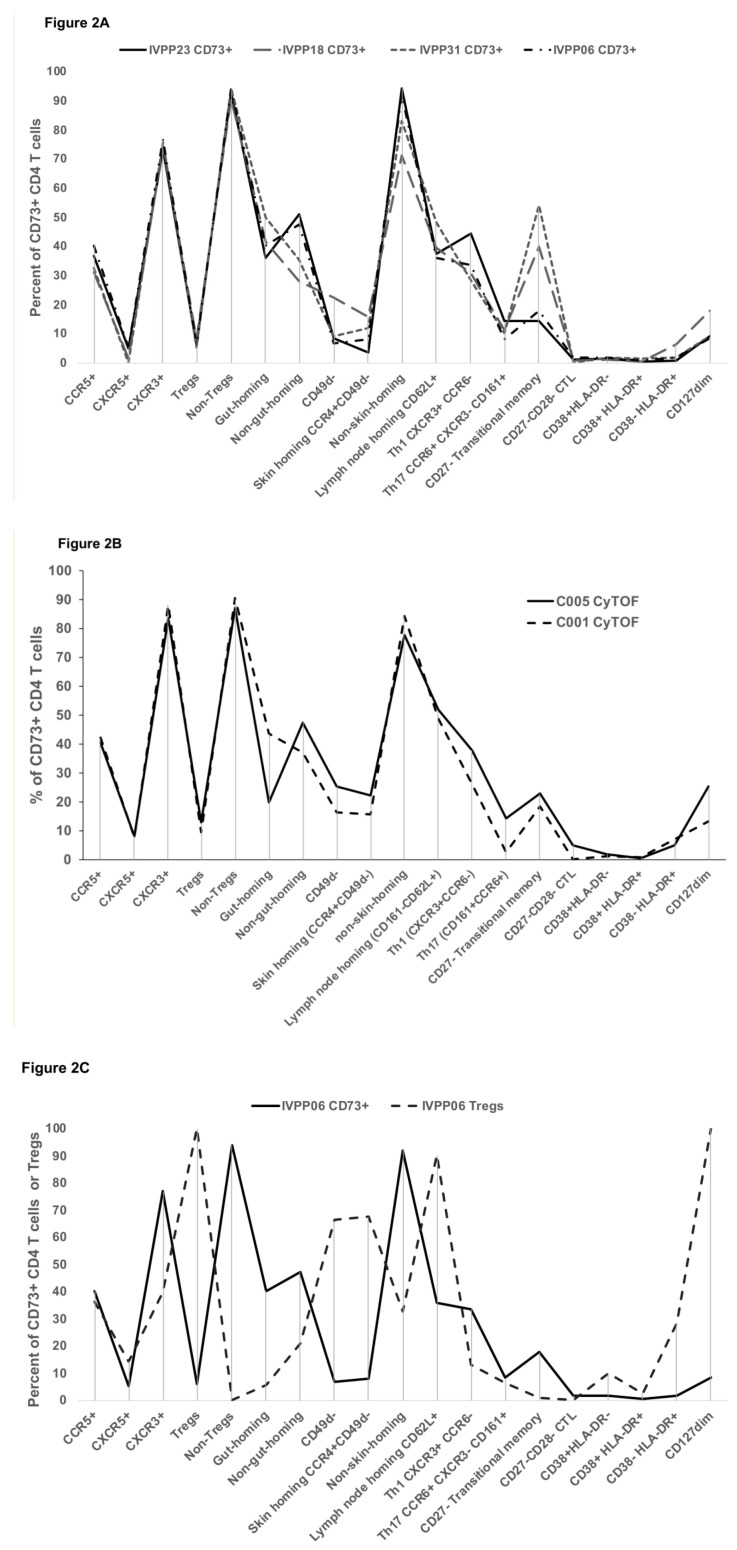

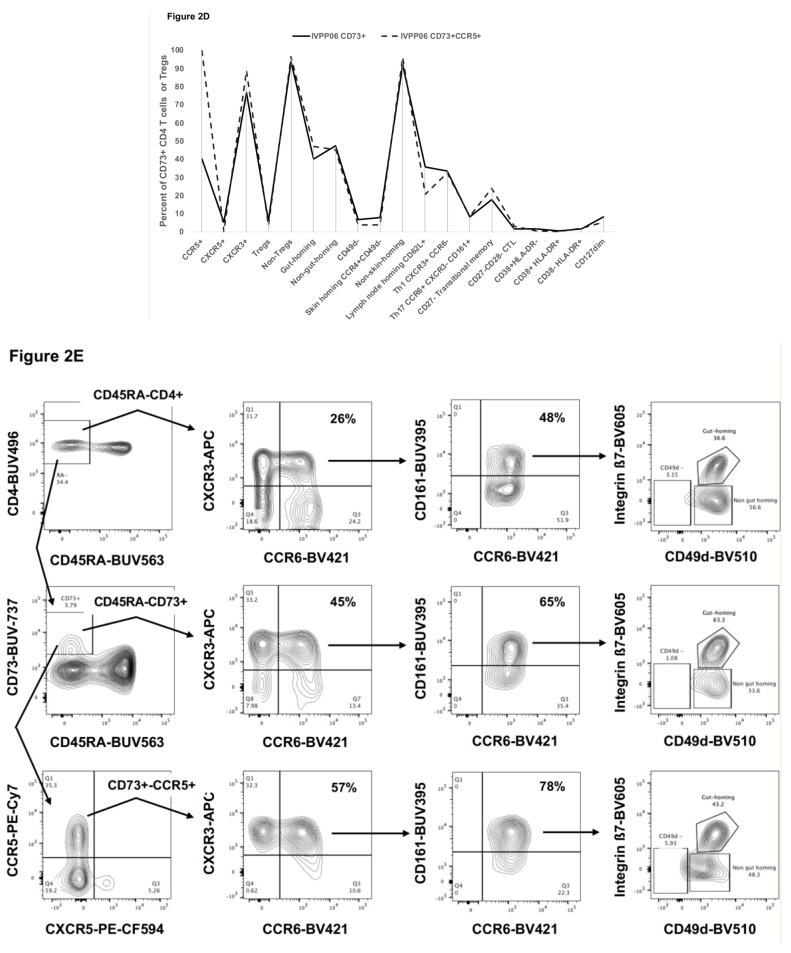
Detailed phenotype of CD73+ memory CD4+ T cells. (**A**) Summary data of FACSymphony 20-colour fluorescence flow cytometry analysis of CD73+ memory CD4+ T cells for *n* = 4 healthy adult controls. (**B**) Summary data of CyTOF 39-parameter mass cytometry analysis of CD73+ memory CD4+ T cells for *n* = 2 healthy adult controls. (**C**) Comparison of 20-colour flow cytometry data for CD73+ CD45RO+ CD4+ T cells versus CD45RO+ Tregs in the same sample shown for one healthy adult control, donor IVPP06. (**D**) Comparison of 20-colour flow cytometry data for CCR5+ CD73+ CD45RO+ CD4+ T cells versus all CD73+ CD45RO+ CD4+ T cells in the same sample from one healthy adult control, donor IVPP06. (**E**) Detailed 20-colour flow cytometry data for the phenotype of CCR5+ CD73+ CD45RO+ CD4+ T cells (lower plots) versus all CD73+ CD45RO+ CD4+ T cells (middle plots), and versus all CD45RO+ CD4+ T cells (upper plots), shown for one donor, IVPP06, from Figure 2A.

The overall expression of important trafficking, activation and differentiation markers was studied on CD73+ memory CD4+ T cells in healthy adult controls by 20-colour fluorescence flow cytometry. Figure 2A shows that CD73+ memory CD4+ T cells exhibit a considerable degree of heterogeneity, with 30–40% being CCR5+, but with few CXCR5+ cells, suggesting skewing towards cell-mediated, rather than humoral, immunity. A high proportion of CD73+ cells are CXCR3+, but not purely Th1-like cells, since many CXCR3+ CD73+ cells also express CCR6. CD73+ CD4+ T cells are predominantly CD49d+, but equally divided between gut-homing CD49d+integrin β7+ and non-gut homing CD49d+integrin β7-negative. In contrast, CD73+ CD4+ T cells contain very few CD49d− negative CCR4+ skin homing cells. Approximately half of CD73+ memory CD4+ T cells are CD62L-negative effector memory cells, including in two individuals an increased proportion of CD27-negative transitional memory cells. Overall, these markers do not indicate either a clear central memory or effector memory phenotype, or a Th1-like (CXCR3+CCR6-) or Th17-like (CXCR3-CCR6+CD161+) phenotype for the CD73+ CD4+ memory cells.

We independently confirmed this CD73+ CD4+ memory T cell phenotype obtained from fluorescence flow cytometry using 39-parameter mass cytometry analysis (Figure 2B). There was very good concordance between the two techniques for samples from the same healthy adult individuals (Supplementary Figure S2B,C).

The high dimensional fluorescence flow cytometry was used to directly compare the same markers on CD73+ CD4+ T cells versus Tregs in the same samples from healthy controls (Figure 2C and Supplementary Figure S2A), which showed significant differences in lymph node-, gut- and skin-homing markers, and especially also in CD127 expression.

The CCR5+ subset of CD73+ CD4+ T cells was analyzed relative to CD73+ cells as a whole (Figure 2D), with little apparent difference. The results in more detail however showed that as memory CD4 T cells were gated first on CD73+ and then on CCR5+ CD73+ cells, the proportion of CXCR3+CCR6+ dual positive cells increased, also with increasing expression of CD161, and included both gut-homing and non-gut homing cells (Figure 2E).

Overall significant differences in the results for phenotyping of CD73+ CD4+ T cells versus CD73-negative CD4+ T cells in Supplementary Figure S1A, and representative flow plots are shown in Supplementary Figure S1B. CD73+ memory CD4+ T cells were predominantly CD26+ (Supplementary Figure S1A,B), which may be functionally important through allowing attachment of adenosine deaminase [24], which could in turn catalyze rapid conversion of adenosine to inosine. In addition, CD73+ CD4+ T cells did not always appear to express cytotoxic effector molecules, including perforin, granzymes or the cytotoxic granule marker TIA-1 (Supplementary Figure S2A, and see below) and therefore are mostly distinct from CD4+ cytotoxic T lymphocytes [25,26,27,28]. Additionally, there appeared to be a slight reduction in CCR7 expression on CD73+ memory CD4+ T cells compared to CD73-negative memory CD4+ T cells, and much lower than naïve CD4+ T cells (Supplementary Figure S4).

### 2.3. Function of CD73+ Memory CD4+ T Cells

**Figure 3:**
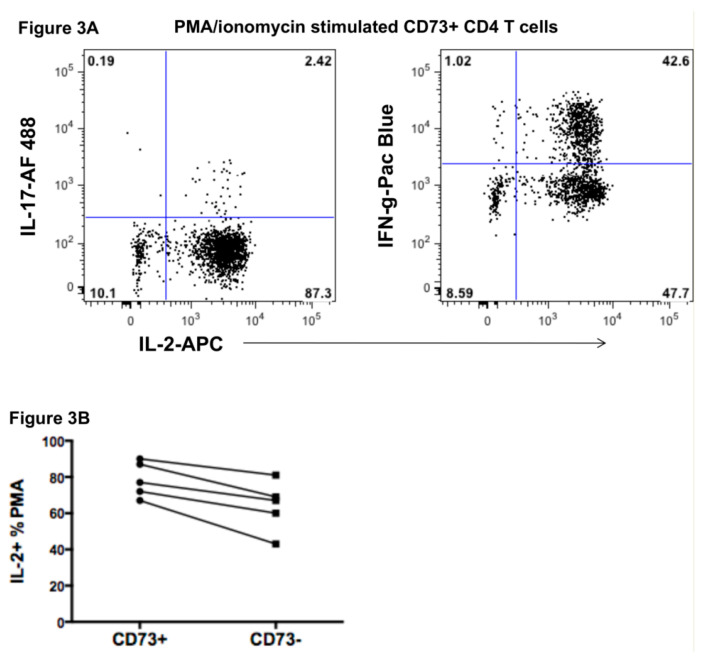
Cytokine production by CD73+ CD45RO+ memory CD4+ T cells. (**A**) Representative flow plots of IL-2, IL-17 and IFN-γ production by CD73+ CD45RO+ memory CD4+ T cells following PMA/ionomycin stimulation in the presence of Brefeldin A. (**B**) Summary data of IL-2 production by CD73+ CD45RO+ memory CD4+ T cells versus CD73-negative CD45RO+ memory CD4+ T cells following PMA/ionomycin stimulation in the presence of brefeldin A.

Important memory T cell functions and lineage commitment were studied in CD73+ CD4+ T cells. Cytokine production by CD73+ CD4+ T cells was measured following phorbol myristate acetate (PMA)/ionomycin stimulation in vitro. The results showed that a very large majority of CD73+ CD4+ T cells produced IL-2, but about half also made IFN-γ, whereas very few made IL-17, consistent with some commitment to the Th1 lineage (Figure 3A). Production of IL-2 was consistently higher by CD73+ CD4+ T cells compared with CD73-negative memory CD4+ T cells, although this did not reach statistical significance (Figure 3B).

**Figure 4:**
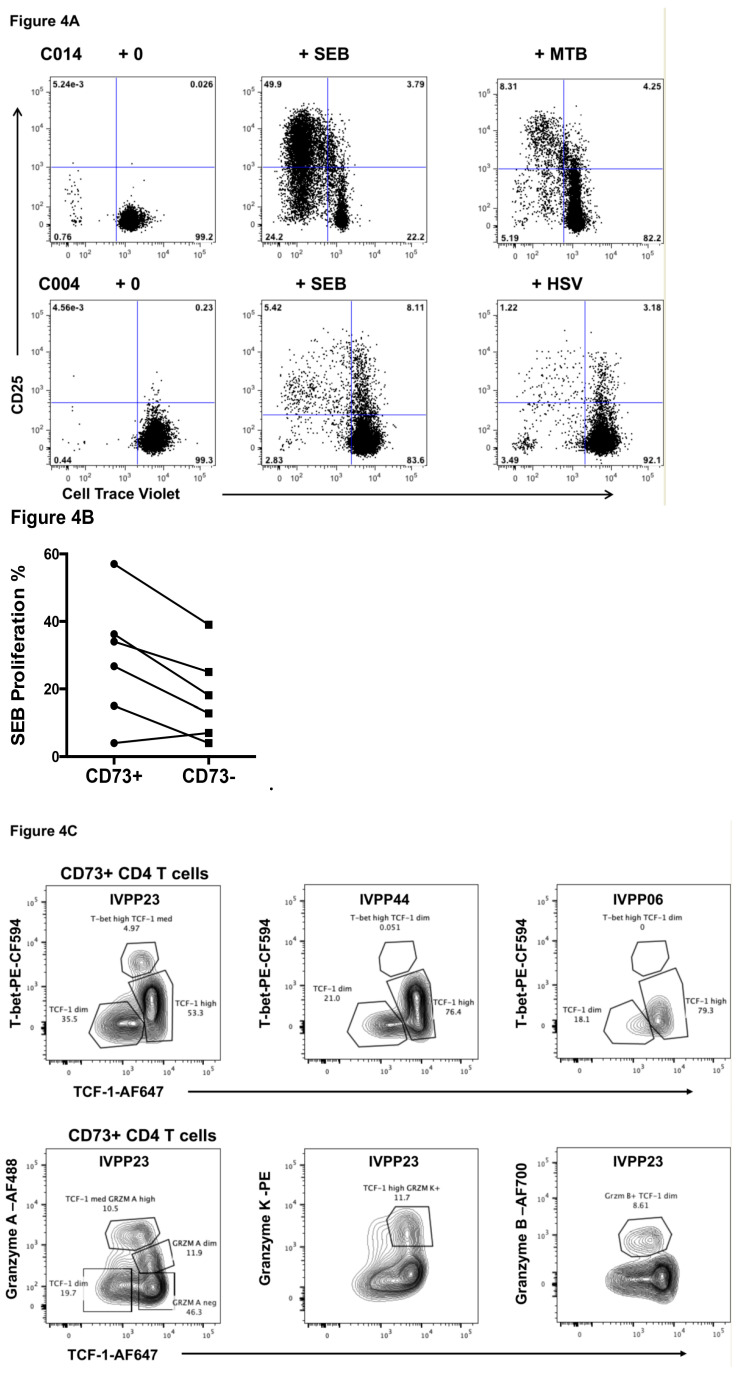

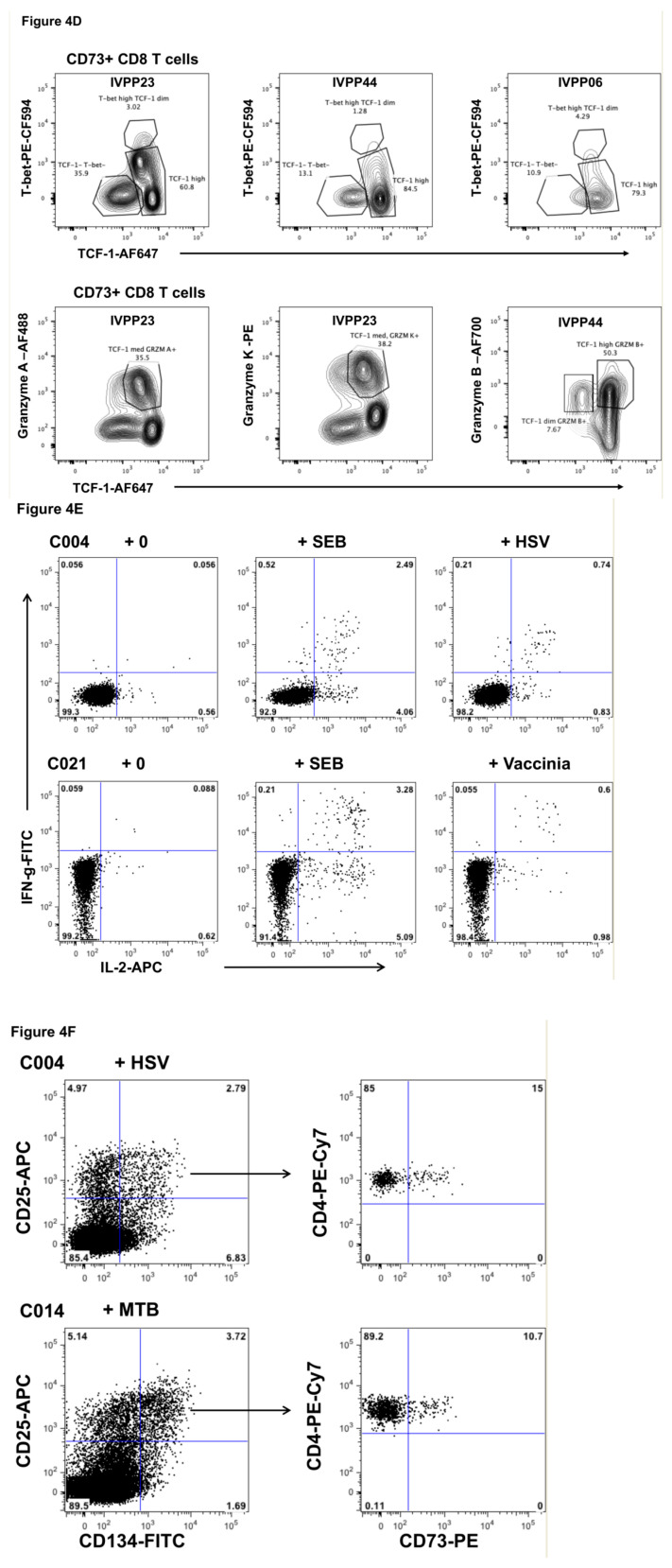

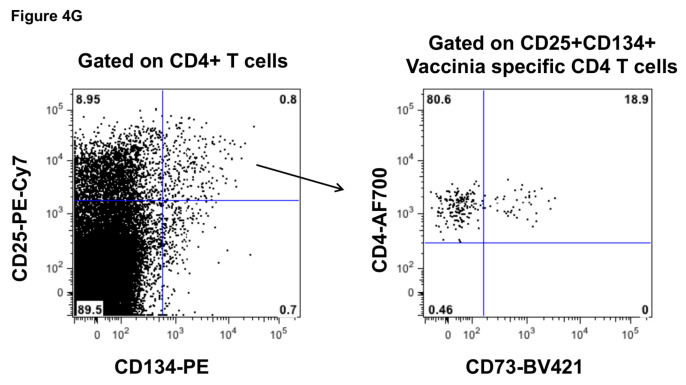
Proliferation of CD73+ CD45RO+ memory CD4+ T cells in vitro. (**A**) Representative flow plots of proliferating CD25^high^ Cell Trace Violet^dim^ (CTV^dim^) CD73+ CD45RO+ memory CD4+ T cells at day 7, in response to mitogen staphylococcal enterotoxin B (SEB), or recall antigens *M. tuberculosis* lysate (MTB) or human simplex virus lysate (HSV) for *n* = 2 healthy adult controls, C014 and C004. (**B**) Summary data of the difference in proliferation in response to the mitogen SEB of CD73+ CD45RO+ memory CD4+ T cells versus CD73-negative CD45RO+ memory CD4+ T cells. (**C**) Transcription and intracellular markers in CD73+ CD45RO+ memory CD4 T cells in peripheral blood from healthy donors. All flowplots were first gated on CD73+ CD45RO+ memory CD4+ T cells. Upper flowplots are from 3 healthy adult donors and show subsets of expression of T-bet and TCF-1. Lower flowplots are from donor IVPP23 and show subsets of cytotoxic molecules, granzymes A, K and B versus TCF-1. (**D**) Transcription and intracellular markers in CD73+ CD8+ T cells in peripheral blood from healthy donors. All flowplots were first gated on CD73+ CD8+ T cells. Upper flowplots are from 3 healthy adult donors and show subsets of expression of T-bet and TCF-1. Lower flowplots are from donor IVPP23 and IVPP44 and show subsets of cytotoxic molecules, granzymes A, K and B versus TCF-1. (**E**) Representative flow plots of intracellular cytokine production by CD73+ CD45RO+ memory CD4+ T cells, in response to mitogen staphylococcal enterotoxin B (SEB), or recall antigens human simplex virus lysate (HSV) or vaccinia virus lysate, for *n* = 2 healthy adult controls C004 and C021. (**F**) Representative flow plots of CD25+CD134+(OX40+) CD4+ T cells, at day 2, in response to recall antigens *M. tuberculosis* lysate (MTB) or human simplex virus lysate (HSV), showing that gated OX40+ CD4 T cells contained detectable CD73+ CD4+ T cells, for *n* = 2 healthy adult controls C004 and C014. (**G**) Representative flow plots of CD25+CD134+(OX40+) CD4 T cells, at day 2, in response to vaccinia virus lysate, showing that gated CD25+OX40+ CD4+ T cells contained detectable CD73+ CD4+ T cells, for healthy adult control C021, representative of 4 experiments.

In vitro proliferative responses of CD73+ memory CD4+ T cells, purified by cell sorting of PBMC from healthy adult controls, were studied using the mitogen SEB as a positive control, and also with a panel of well-defined recall antigens for human memory CD4+ T cells (Figure 4A). Isolated CD73+ memory CD4+ T cells did not proliferate or upregulate CD25 in the absence of stimulation, but proliferated vigorously and upregulated CD25 in response to SEB in five out of six experiments, with a higher proliferation rate than the corresponding CD73-negative memory CD4+ T cells (Figure 4B). CD73+ memory CD4+ T cells proliferated in response to recall antigens, including *M. tuberculosis* (Mtb) or herpes simplex virus (HSV) lysates, respectively (Figure 4A, right flowplots).

CD73+ CD4+ T cells are memory cells that are quiescent, lacking markers of activation in vivo (Figure 1B and Figure 2A, and see below), yet readily proliferate upon antigenic and mitogenic stimulation. Recently, these characteristics in CD8+ memory cells in murine models have been associated with high expression of the transcription factor TCF-1 [29], and downregulation of the coinhibitory receptor PD-1 [30]. Therefore, we examined expression of TCF-1 in CD73+ CD4+ T cells, and found that a large proportion were TCF-1^high^ (Figure 4C).

The CD73+ CD4+ T cells could be further subdivided into subsets based on expression of T-bet and granzymes A, K and B (Figure 4C), in healthy adults who are CMV seropositive and have CD4 cytotoxic T lymphocytes [26]. Similarly, in these individuals, a subset of the CD73+ CD4+ T cells also coexpressed the cytotoxic granule marker TIA-1 (not shown).

CD73+ CD8+ T cells were also heterogeneous with respect to T-bet, TCF-1 and cytotoxic effector phenotypes (Figure 4D).

Overall, the presence of TCF-1^high^ CD73+ CD4 T cells is consistent with their including effector cells capable of further proliferation [30]. Importantly, the promoter for the gene encoding CD73 contains a TCF-1 consensus binding site, and transfection with TCF-1 increases its promoter activity [31]. Additionally, consistent with the relatively high proliferative capacity, CD73+ is clearly reciprocally expressed on CD4 T cells with the checkpoint inhibitors TIGIT, PD-1 and TIM-3 [32] (see Figure 1B).

Antigen-specific CD73+ memory CD4+ T cells were also detected using IFN-γ and IL-2 production in response to HSV and vaccinia lysate stimulation (Figure 4E). Similarly, the CD25/CD134 (“OX40”) assay detects antigen-specific CD4+ T cells through induced coexpression of CD25 and CD134 after 40–48 h incubation with antigen [33]. Addition of anti-CD73 mAb to the OX40 assay staining panel shows that HSV- and Mtb-specific CD4+ T cells include CD73+ cells (Figure 4F).

Interestingly, we detected vaccinia-specific CD4+ T cells in four subjects where the date of inoculation is known (from 1 to 10 years previously). We found that these subjects had CD73+ vaccinia-specific CD4+ T cells, as measured by either intracellular cytokine or OX40 assays (Figure 4G). For three subjects, the CD73+ percentages of vaccinia-specific CD4+ T cells was 17.5%, 18.9%, and 14.8%, respectively, and for the donor inoculated 10 years earlier, the CD73+ percentage of vaccinia-specific IFN-γ+IL-2+ CD4+ T cells was 70%.

### 2.4. Transcriptomics of CD73+ Memory CD4+ T Cells

We used microarray analysis to study the transcriptome of CD73+ CD4+ and CD8+ T cells versus CD73-negative CD4+ and CD8+ T cells, purified by cell sorting from three donors, and the highest and lowest differentially expressed genes are shown in Supplementary Tables S2 and S3. A very highly differentially expressed gene in CD73+ T cells compared to CD73-negative T cells was the natural killer-tumor recognition sequence (*NKTR*), consistent with the finding of a subset of CD73+ with a CTL phenotype (Figure 4C). However, CD73+ T cells overall had less cytotoxic effector molecules mRNA transcripts, especially Granzyme H (Supplementary Tables S2 and S3).

The transcriptomic data showing reduced *CXCR5, CCR7, CCR4, HLA-DR, TIGIT* and *CD25* gene expression by purified CD73+ memory CD4+ T cells, compared to CD73-negative memory CD4+ T cells (Supplementary Table S2) was consistent with observations of their reduced protein expression by flow cytometry, (see Figure 1B and Figure 2A and Supplementary Figure S1A–C).

*BTAF1*, which encodes the B-TFIID TATA-Box Binding Protein Associated Factor 1, was the only transcription factor with >2-fold differential expression in CD73+ CD4+ and CD73+CD8+ T cells (9.6-fold and 4.1-fold increases compared to CD73-negative cells, respectively). BTAF1 and TATA binding protein (TBP) together make up the B-TFIID complex, which regulates gene expression, and therefore it is possible that this complex differentially regulates CD73 expression.

### 2.5. Response of CD73+ Memory CD4+ T Cells to Incubation with IL-7 and IL-2

**Figure 5:**
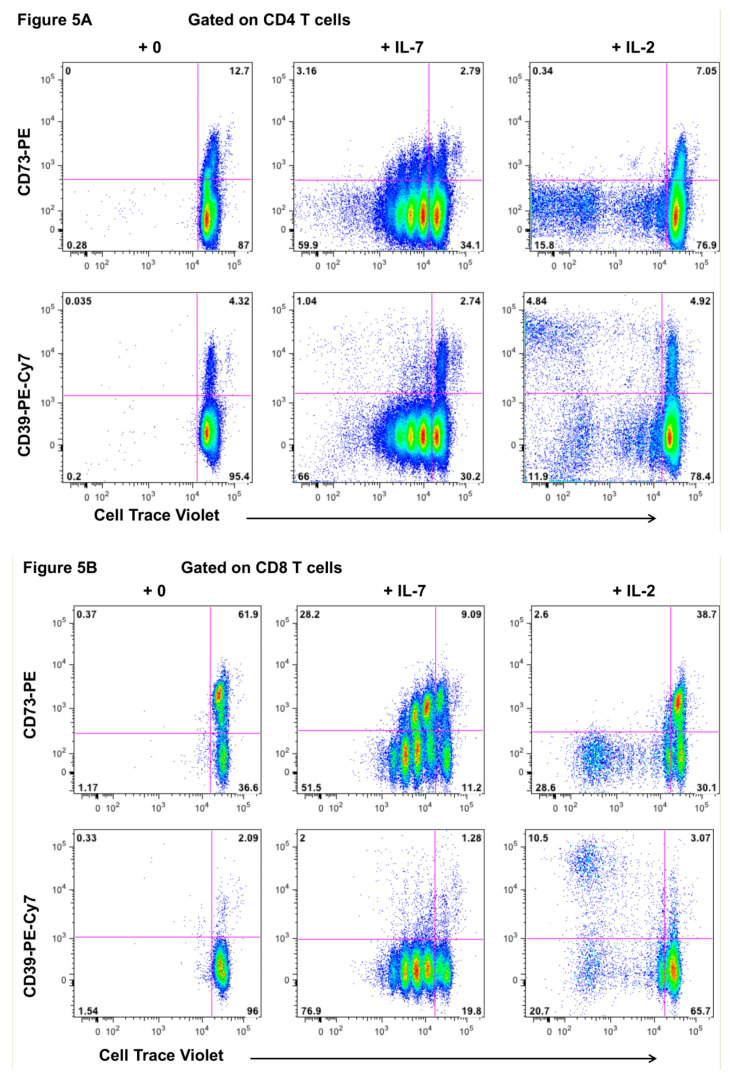

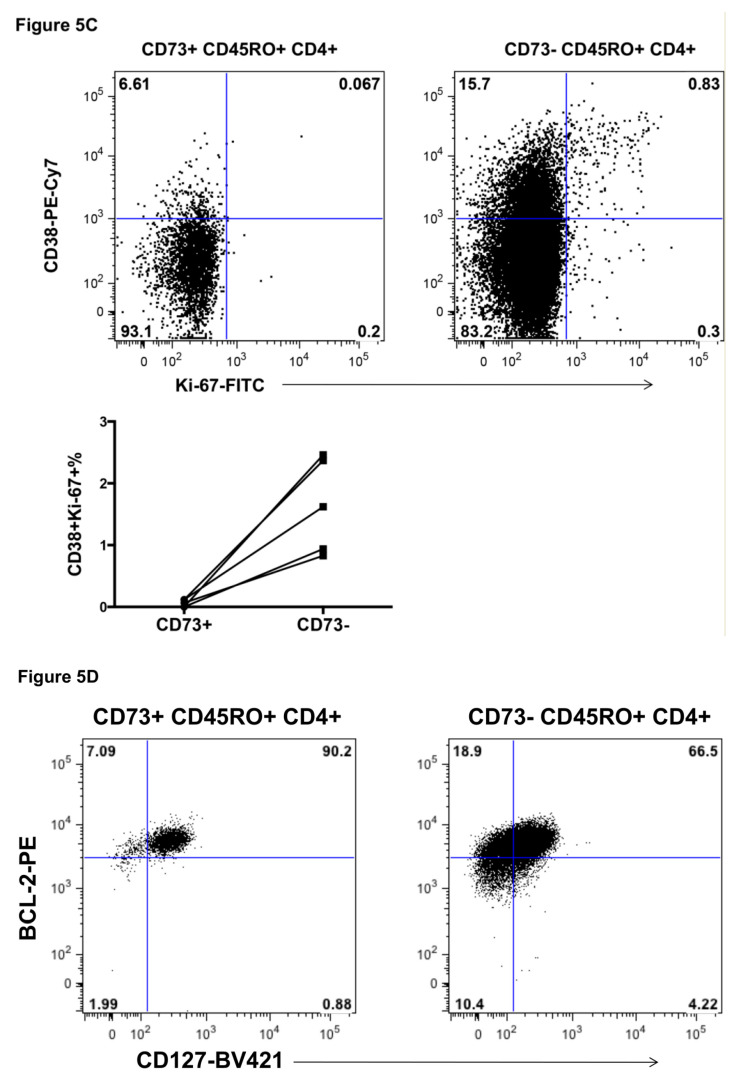
Proliferation of CD73+ CD4+ T cells in vitro, but not in vivo. (**A**) Representative flow plots showing the proliferation of CD73+ CD4+ T cells (top row) and CD39+ CD4+ T cells (bottom row) within PBMC after 7 days culture with IL-7 or IL-2. Results are representative of 3 experiments. (**B**) Representative flow plots showing the proliferation of CD73+ CD8+ T cells (top row) and CD39+ CD8+ T cells (bottom row) within PBMC after 7 days culture with IL-7 or IL-2. Results are representative of 3 experiments. (**C**) Representative flow plots of CD73+ CD4+ T cells in fresh peripheral blood, showing that CD73+ CD45RO+ memory CD4+ T cells have very low levels of the activation antigen CD38 and an intracellular marker of cell cycling Ki-67, compared CD73-negative CD45RO+ memory CD4+ T cells. Bottom graph shows summary data from *n* = 5 healthy adults. (**D**) Representative flow plots showing coexpression of CD127 and antiapoptotic intracellular marker Bcl-2 in CD73+ CD45RO+ memory CD4+ T cells compared to CD73-negative CD45RO+ memory CD4+ T cells. Results are representative of 3 experiments.

Since CD73+ memory CD4+ T cells express the alpha chain of the IL-7 receptor, CD127, but not the alpha chain of the IL-2 receptor, CD25, in PBMC (Figure 1), we tested the response of these cells to culture in vitro with IL-7 or IL-2. We detected proliferating CD73+ CD4+ T cells after 7 days within PBMC cultured with IL-7, whereas IL-2 alone did not result in proliferation of CD73+ CD4+ T cells (Figure 5A, top row flowplots). Conversely, CD39+ was highly expressed on CD4+ T cells, which proliferated in IL-2 cultures, but was not expressed on cells that proliferated in IL-7 cultures (Figure 5A, lower row flowplots), consistent with CD39+ CD4+ memory T cells in PBMC expressing CD25, but not CD127. Analogous results were observed for CD73+ CD8+ T cells, which proliferated in response to IL-7, but not IL-2 (Figure 5B).

To investigate whether CD73+ memory CD4+ T cells were cycling in vivo, we used the activation marker CD38 in conjunction with the nuclear marker of recent proliferation, Ki-67 (Figure 5C). In healthy adult controls, Ki-67+ CD73+ memory CD4+ T cells were virtually undetectable. In contrast 1–3% of CD73-negative memory CD4+ T cells were Ki-67+. Furthermore, the CD73+ memory CD4+ T cells were almost all Bcl-2high in conjunction with CD127 expression (Figure 5D). Together these results were consistent with CD73+ CD4+ T cells belonging to the long-lived resting memory T cell compartment.

### 2.6. Effect of HIV-1 Infection on CD73+ Memory CD4+ T Cells

**Figure 6:**
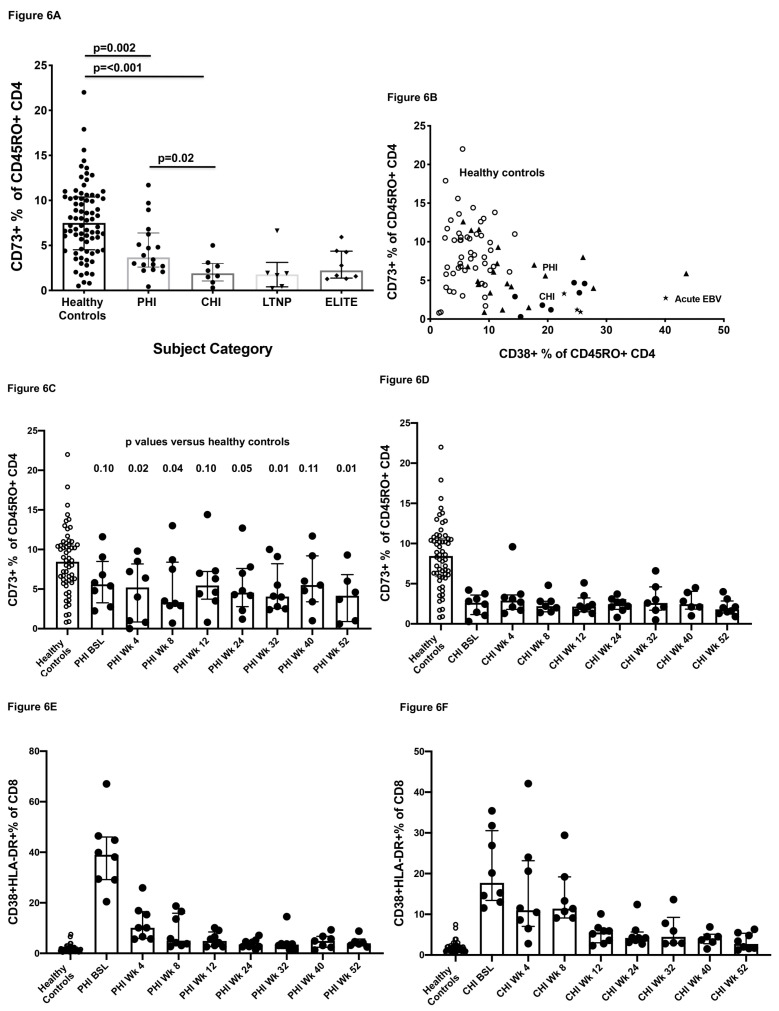

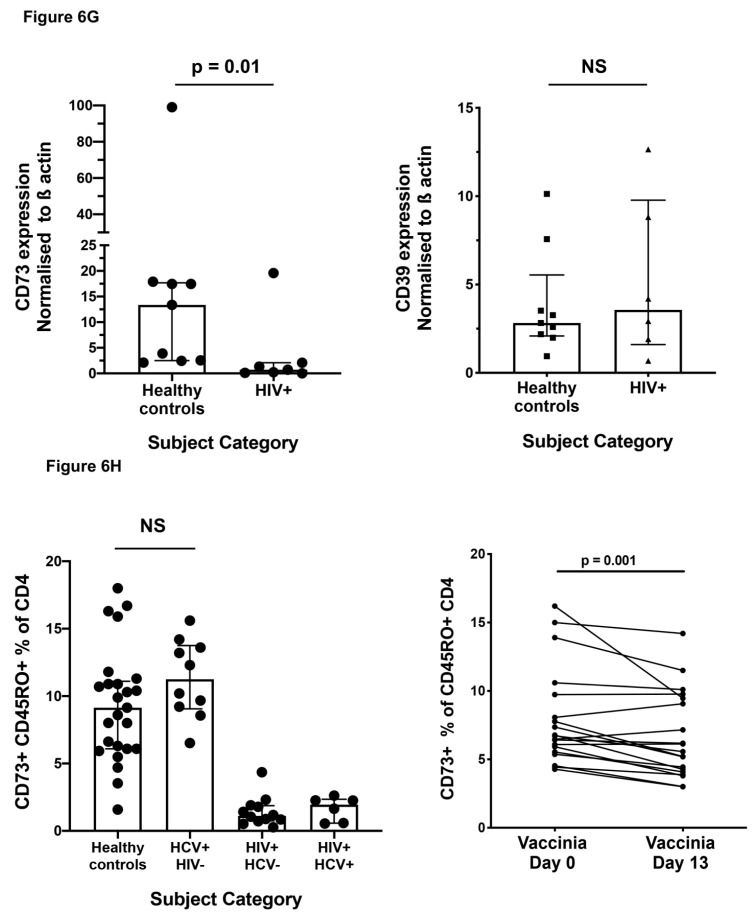
Progressive loss of CD73+ CD45RO+ CD4+ memory cells during HIV-1 infection. (**A**) Comparison of CD73+ cells as percentage of CD45RO+ memory CD4+ T cells in healthy adult controls, primary HIV-1 infection (PHI) subjects, chronic HIV-1 infection (CHI) subjects, HIV+ long-term survivors (LTNP) and HIV+ Elite controllers (Elite). (**B**) Graph of CD73+ CD45RO+ memory cells as percentage of CD4+ T cells versus CD38+ CD45RO+ memory cells as percentage of CD4+ T cells in the same samples, in healthy adult controls (small circles), compared to PHI subjects at presentation (triangles), CHI subjects (large solid circles) and acute EBV subjects (stars). (**C**) Prospective longitudinal data of CD73+ CD45RO+ memory cells as percentage of CD4+ T cells in *n* = 8 PHI subjects commencing antiretroviral therapy including integrase inhibitor. Results are from real-time analysis from baseline (BSL) to week 52. *p* values are for comparison to healthy adult control values from samples run over the same time period using the same method. (**D**) Prospective longitudinal data of CD73+ CD45RO+ memory cells as percentage of CD4+ T cells in *n* = 8 CHI subjects commencing antiretroviral therapy including integrase inhibitor, compared to healthy adult control values from samples run over the same time period using the same method. Results are from real-time analysis from baseline (BSL) to week 52. (**E**) Prospective longitudinal data of activated CD38+HLA-DR+ as a percentage of CD8+ T cells in *n* = 8 PHI subjects commencing antiretroviral therapy including an integrase inhibitor. Results are from the same real-time analysis from baseline (BSL) to week 52 as for Figure 2C, compared to healthy adult control values from samples run over the same time period using the same method. (**F**) Prospective longitudinal data of activated CD38+HLA-DR+ as percentage of CD8+ T cells in *n* = 8 CHI subjects commencing antiretroviral therapy including integrase inhibitor. Results are from the same real-time analysis from baseline (BSL) to week 52 as for Figure 2D, compared to healthy adult control values from samples run over the same time period using the same method. (**G**) Decreased expression of mRNA for CD73 (*NT5E*), by quantitative RT-PCR of purified CD4+ T cells from HIV+ subjects, compared to healthy controls (left graph), and mRNA for CD39 (*ENTPD1*), which was not significantly different, in purified CD4+ T cells from HIV+ subjects, compared to healthy controls (right graph). (**H**) Comparison of CD73+ cells as percentage of CD45RO+ memory CD4+ T cells in healthy adult controls, compared to HIV+ subjects, hepatitis C virus (HCV) infected subjects and HIV and HCV-coinfected subjects (left graph), and for healthy adults at baseline and at day 13 post-inoculation with vaccinia virus (right graph).

The attributes of CD73+ CD4+ T cells, particularly IL-2 production and proliferative capacity in response to recall antigens, are notably deficient in the CD4+ T cell function of the vast majority of subjects with HIV-1 infection soon after infection [34]. Therefore, we compared these cells in healthy adult controls versus subjects with primary HIV-1 infection, chronic HIV-1 infection, long-term HIV+ non-progressors (LTNP) and elite HIV-controllers, respectively (Figure 6A and representative flow plots in Supplementary Figure S3). The results show that there was a significant reduction in the proportion of CD73+ memory CD4+ T cells at the earliest stages of primary HIV-1 infection, and a further reduction in subjects with chronic HIV-1 infection. Interestingly, even LTNP and elite controllers in this study also showed a significant decrease in the proportion of CD73+ memory CD4+ T cells, but by definition, these subjects retained relatively high CD4+ T cell counts, and therefore, the decrease will not be as profound numerically.

As previously described [12], there was an inverse relationship between the CD73+% of memory CD4 T cells and the activated CD38+% of memory CD4+ T cells in the cross-sectional data for HIV+ patients at baseline, compared to healthy controls (Figure 6B), prior to commencing ART. However, acute EBV infection also was associated with a decrease in the proportion of CD73+ CD4+ T cells and an increase in activated CD38+ CD4+ T cells (Figure 6B).

Two cohorts, one of primary HIV-1 infection (PHI) and one of chronic HIV-1 infection (CHI) subjects were followed prospectively over 52 weeks, following commencement of antiretroviral therapy (ART) including Raltegravir. The results show clearly that the proportion of CD73+ memory CD4+ T cells did not normalize following 52 weeks of therapy in either cohort (Figure 6C,D), but in contrast the levels of activation rapidly tended towards normalization in both (Figure 6E,F), in line with the rapid decrease in plasma viral load in these patients [35]. 

These findings for CD73+ CD4+ T cells were replicated using cryopreserved PBMC from separate cohorts of treatment-naïve late stage chronic HIV-infected subjects from the PRIRIS study [36] (Supplementary Figure S5) and the OZCOMBO study [37].

Quantitative RT-PCR was used to show that the decrease in the proportion of CD73+ CD4+ T cells was associated with a decrease in the levels of CD73 mRNA in CD4+ T cells, but CD39 mRNA was not decreased (Figure 6G), suggesting that the reduced frequency of CD73+ cells detected by flow cytometry was not simply due to intracellular sequestration or shedding of the CD73 protein [38].

Another chronic viral infection, HCV, did not lead to reduction in the proportion of CD73+ memory CD4+ T cells compared to healthy controls (Figure 6H). Only HCV-infected subjects who were HIV-coinfected exhibited a reduction in the proportion of CD73+ memory CD4+ T cells (Figure 6H). Similarly, we also studied healthy volunteers undergoing vaccinia inoculation as a comparator model of a primary viral infection. These vaccines (*n* = 20) exhibited a transient peak in CD38+ memory CD4+ effector T cells at around day 13 post-inoculation [39], and there was also a slight, but significant, decrease in CD73+% of CD45RO+ CD4+ T cells for *n* = 20 volunteers (Figure 6H). The magnitude of the change was not as great as the reduction observed when comparing primary HIV-1 infection subjects with healthy adults. This was despite the fact that the relative increase in CD38+ CD4+ T cells was comparable between the vaccinia and primary HIV-1 infection cohorts, as previously reported [27].

CD73+ CD8+ T cells were also greatly reduced as a proportion of CD8+ T cells in the PHI and CHI subjects, and HIV+/HCV coinfected and acute EBV infection patients, but not HCV only patients (Supplementary Figure S4).

### 2.7. CD73+ Memory CD4+ T Cells in Gut Biopsies

Since a significant subset of CD73+ CD4+ T cells expressed the gut-homing markers integrins α4β7 (see above), we examined single suspensions derived from gut biopsies from 14 HIV-uninfected volunteers and 13 HIV-infected volunteers, undergoing colonoscopy, for the presence of CD73+ CD4+ T cells.

**Figure 7:**
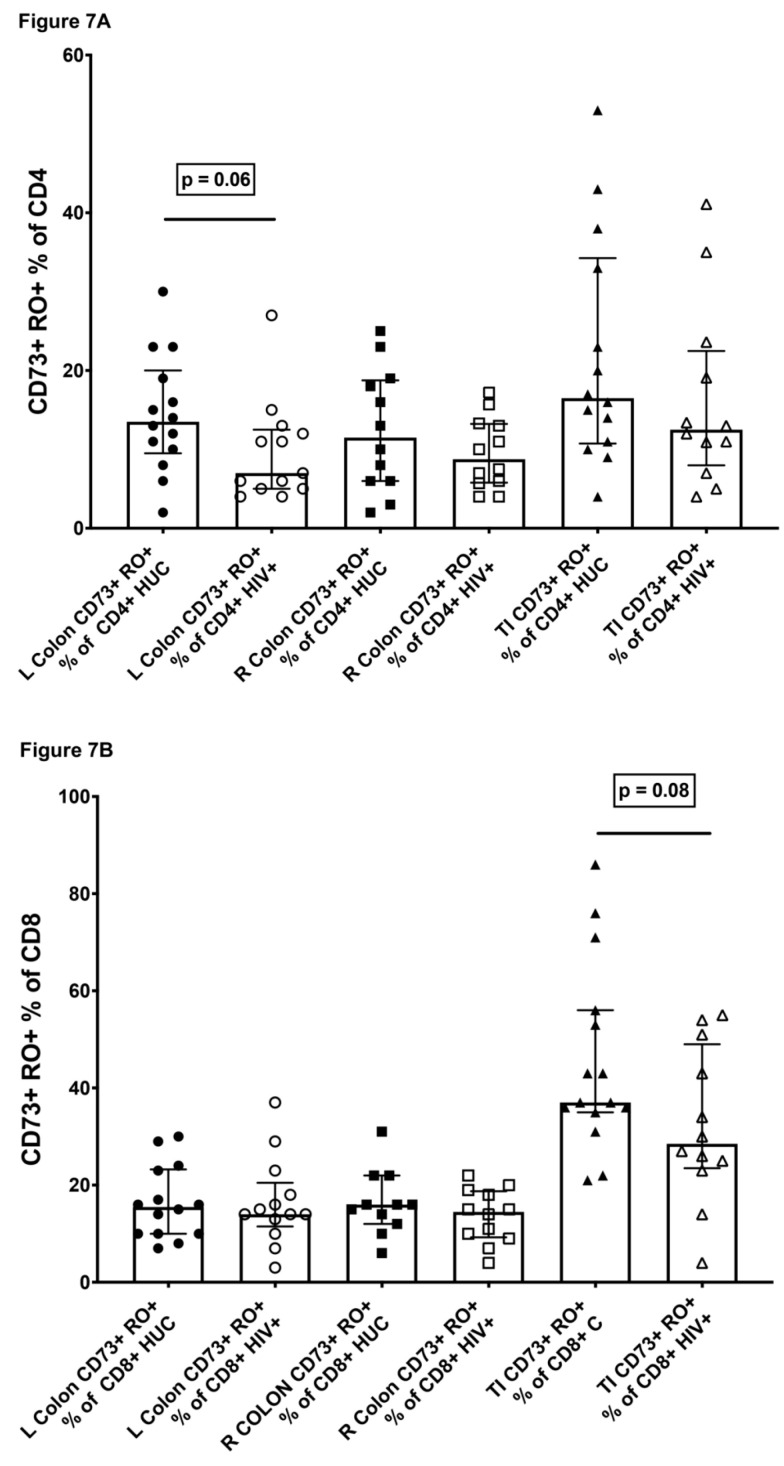

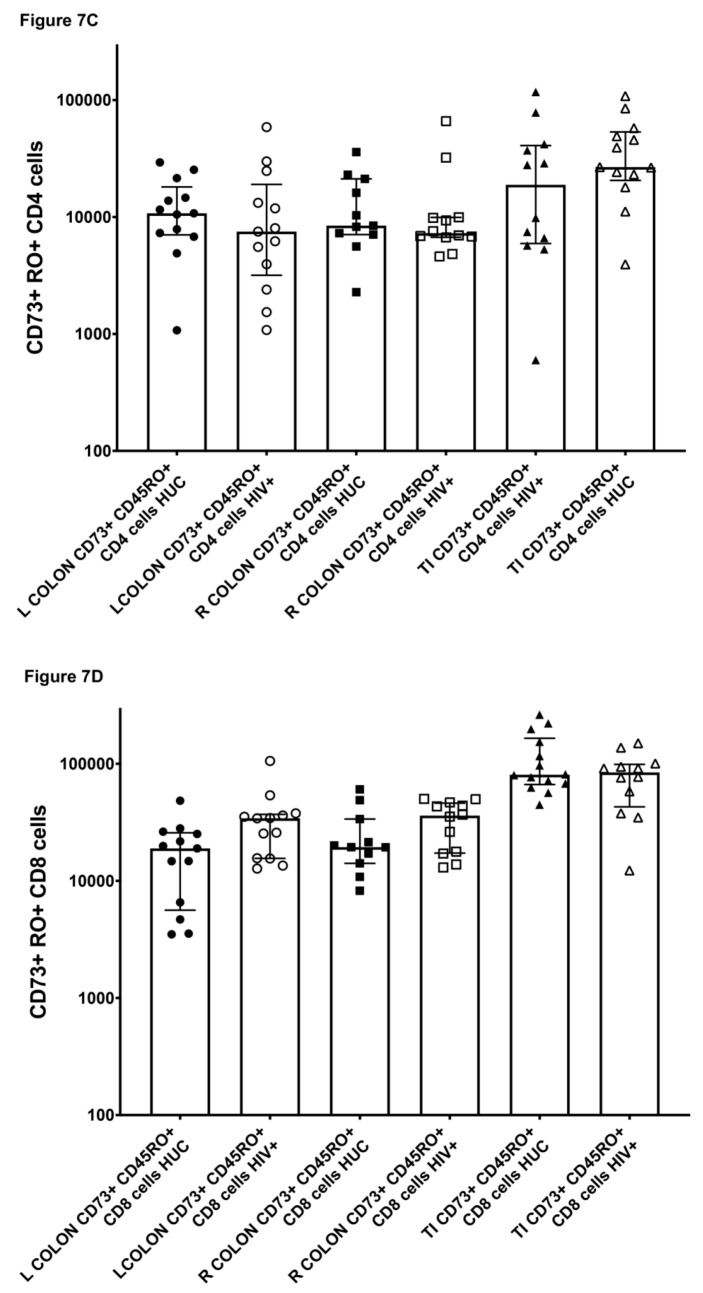
CD73+ CD45RO+ memory CD4+ T cells in gut biopsies. (**A**) CD73+ CD45RO+ memory cells as a percentage of CD4+ T cells in single cell suspensions prepared from left colon (LC), right colon (RC) or terminal ileum (TI), from subjects that were HIV-uninfected controls (HUC), versus HIV+ subjects on ART (HIV+). (**B**) CD73+ CD45RO+ memory cells as percentage of CD8+ T cells in single cell suspensions prepared from left colon (LC), right colon (RC) or terminal ileum (TI), from subjects that were HIV-uninfected controls (HUC), versus HIV+ subjects on ART (HIV+). (**C**) Absolute cell counts of CD73+ CD45RO+ memory CD4+ T cells in single cell suspensions prepared from left colon (LC), right colon (RC) or terminal ileum (TI), from subjects that were HIV-uninfected controls (HUC), versus HIV+ subjects on ART (HIV+). (**D**) Absolute cell counts of CD73+ CD45RO+ memory CD8+ T cells in single cell suspensions prepared from left colon (LC), right colon (RC) or terminal ileum (TI), from subjects that were HIV-uninfected controls (HUC), versus HIV+ subjects on ART (HIV+).

The results show that in gut biopsy samples from the left colon, right colon and terminal ileum, from HIV-uninfected adult controls, CD73+ CD4+ T cells were readily detectable, with medians typically in the range 10–20% of CD4+ T cells (Figure 7A). However, particularly for biopsies from terminal ileum, the individual CD73+ percentages exhibited a wide range from 5 to 50% of CD4+ T cells. This may be due to whether biopsies included samples from Peyer’s patches or not. The proportions of CD73+ CD8+ T cells were in general higher than for CD73+ CD4+ T cells, particularly in the terminal ileum, where the median CD73+ proportion reached 37% of CD8+ T cells (Figure 7B).

In gut biopsy samples from HIV-infected, ART-treated patients, we did not observe the same degree of reduction of the proportion of CD73+ memory CD4+ T cells that we found in peripheral blood (Figure 7A, compared with Figure 6A). Furthermore, we accurately counted the number of CD4+ T cells recovered from the biopsies and did not find in these patients any dramatic decrease in CD4+ T cells in general [40] or CD73+ memory CD4+ T cells in particular (Figure 7C), nor CD73+ memory CD8 T cells (Figure 7D). Since large variability was observed between individual subjects, we also normalized lymphocyte counts using the number of epithelial cells recovered in the cells suspensions, to account for possible differences in biopsy sizes or efficiency of tissue digestion, as previously described [40], and very similar trends were observed (Supplementary Figure S6).

### 2.8. HIV-Infection of CD73+ CD4+ T Cells

**Figure 8:**
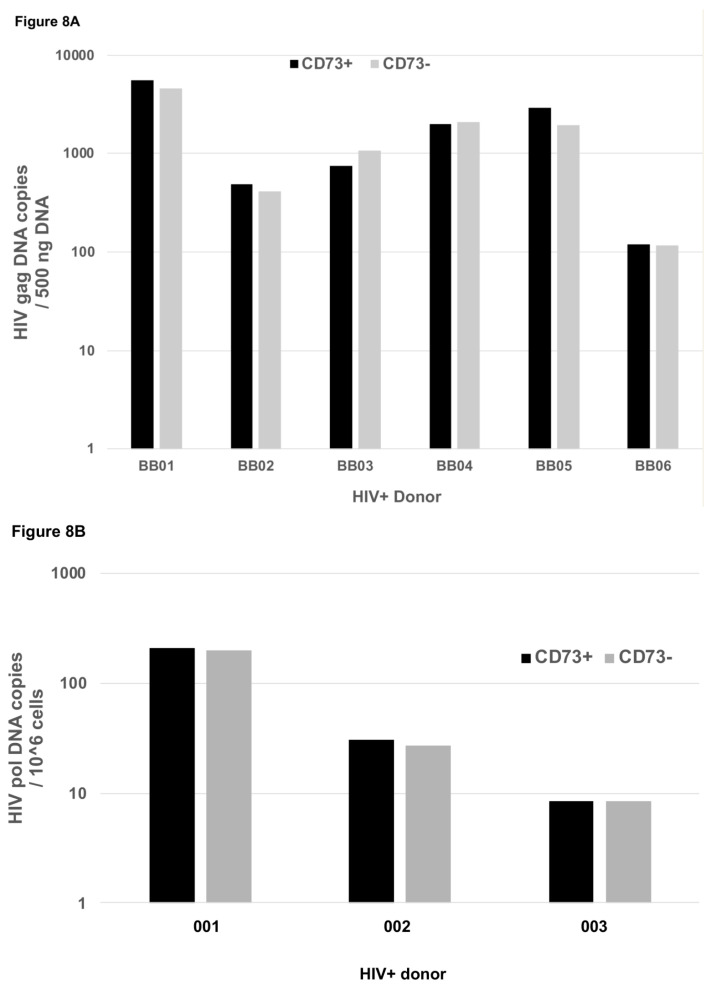
HIV DNA infection of CD73+ CD4+ T cells in peripheral blood. (**A**) HIV DNA *gag* copies (normalized to 500 ng DNA) were measured by quantitative real time PCR in purified CD73+ CD4+ T cells from cryopreserved leukapheresis product, from untreated HIV+ subjects. The graph shows the ratio of copy number in highly purified CD73+ CD4+ T cells compared to the copy number in total CD4+ T cells for *n* = 6 individuals. (**B**) HIV DNA *pol* copies (normalized to 10^6^ cells) were measured by quantitative real time PCR in purified CD73+ CD4+ T cells from cryopreserved PBMC, from HIV+ subjects on long term ART. The graph shows the copy number in highly purified CD73+ CD4+ T cells compared to the copy number in CD73-negative CD4+ T cells for *n* = 3 individuals.

Given that a significant subset of CD73+ CD4+ T cells expressed the HIV-1 co-receptor CCR5, we sought to examine whether these cells, in HIV+ donor peripheral blood, had been infected in vivo and harbored HIV DNA. The reduced proportions of CD73+ CD4+ T cells in chronic HIV-1 infection meant that we needed to use cryopreserved PBMC obtained by leukopheresis from a cohort of untreated patients [41], to sort CD73+ and CD73-negative subsets of memory CD4+ T cells. The results show that purified CD73+ CD4+ T cells contained HIV DNA (Figure 8A), consistent with their expression of CCR5. There was no significant difference, by paired comparison, for the relative amounts of HIV DNA between CD73+ versus CD73-negative memory CD4+ T cell subsets (ranges: 120–5567 versus 115–4540 copies/500 ng DNA, respectively; Figure 8A).

We further investigated HIV DNA in CD73+ memory CD4+ T cells from HIV+ subjects who had been on fully suppressive ART for at least 2 years (Figure 8B) [42]. Again, the levels of HIV DNA detected in CD73+ memory CD4+ T cells in PBMC was not significantly different, by paired comparison, to the levels found in CD73-negative memory CD4+ T cells (ranges: 8.5–212 versus 8.5–200 copies/10^6^ cells, respectively; Figure 8B).

## 3. Discussion

Our results show that CD73+ CD4+ T lymphocytes phenotypically represent a subset of resting memory cells that can proliferate in vitro in response to the common human CD4+ T cell recall antigens Mtb, Herpes Simplex Virus (HSV), CMV and vaccinia virus. Importantly, we found a significant reduction in CD73+ CD4+ T cells in early primary HIV-1 infection, with these cells further reduced during chronic HIV-1 infection, confirming and extending previous results [6,10]. Prospective longitudinal studies showed that the loss of CD73+ CD4+ T cells in peripheral blood was not rectified by ART, even if commenced during primary infection, suggesting this reduction was not due to cellular redistribution during viremia, and that they are not easily replaced once lost, although their robust proliferation to IL-7 stimulation in vitro suggests they can undergo homeostatic proliferation.

To date, studies of lymphocyte expression of the ecto-enzyme CD73 have focused on its enzymatic conversion of AMP to adenosine, which is thought to be an important immunomodulatory Treg feedback mechanism to limit immune responses [2,4]. However, the absolute requirement of this enzyme for Treg function is unclear, with CD73 knockout mice having a relatively normal immune phenotype [43] compared to, for example, deletion of the well-defined Treg-associated inhibitory molecule, CTLA-4 [44]. In fact, rare human individuals have now been identified with loss of function mutations in the NT5E gene, leading to a lack of CD73 protein and enzymatic activity, who are characterized by arterial calcification due to vascular dysplasia, but with no reported manifestations of autoimmunity (reviewed in [45]). Furthermore, it is well described that in HIV+ subjects commencing ART, either during primary or chronic HIV-1 infection, CD4+ and CD8+ T cell activation is progressively reduced [46,47], but this occurs in the absence of any reconstitution of CD73+ CD4+ T cells, as previously described for ART commenced during CHI [12], and confirmed prospectively in this study for ART commenced in both CHI and PHI.

Instead, there is evidence that CD73 is involved in lymphocyte migration, particularly into lymph nodes via afferent lymphatics rather than high endothelial venules [15,16]. Our detailed phenotypic analysis of CD73+ CD4+ memory T cells revealed they express a distinct pattern of trafficking molecules, largely lacking CD62L and with slightly reduced expression of CCR7, and relatively high coexpression of CXCR3 and CCR6, consistent with the idea that CD73+ CD4+ T cells undertake a different pathway of immunosurveillance to the more classical central memory cells that extravasate via HEV into peripheral lymphoid tissue.

In contrast, Tregs in the same individuals highly expressed CD62L and CCR7, and have a higher frequency in the inguinal lymph node fine needle biopsies from healthy controls, compared to their peripheral blood [23], whereas in this study CD73+ CD4+ T cell frequency was approximately half in inguinal lymph nodes compared to peripheral blood. Additionally, Tregs were predominantly CD49d-negative and CCR4+, associated with skin-homing, whereas CD73+ CD4+ T cells were predominantly CD49d+ and CCR4-negative. In particular, very few Tregs in peripheral blood were gut-homing CD49d+integrin ß7+, whereas approximately half of CD73+ CD4+ T cells had a gut-homing CD49d+integrin ß7+ phenotype. Furthermore, CD73+ CD4+ T cells were observed in CSF at frequencies comparable to peripheral blood, whereas Treg frequencies were much lower. Altogether, based on the phenotypes in peripheral blood, combined with direct measurements in tissues, Tregs and CD73+ CD4+ T cells have quite disparate patterns of circulation.

Similarly, CD73+ CD4+ T cells predominantly lack CXCR5, suggesting that they will not specifically migrate to B cell areas in lymph nodes, and presumably not interact directly with CD39+ B cells. Instead, relatively high expression of CCR5 and CXCR3 on CD73+ CD4+ T cells is indicative of a role in cell-mediated immunity. We however did not confirm a previous result of high expression of IL-17 by CD73+ CD4+ T cells [5], possibly because we used PMA/Ionomycin stimulation of cells from healthy adult controls, compared to anti-CD3/CD28 stimulation of cells from inflammatory bowel disease patients in the previous study.

We were able to confirm that the relatively high proportion of circulating CD73+ CD4+ T cells that coexpressed integrin α4β7+ indeed reflected the presence of CD73+ CD4+ T cells in gut-associated lymphoid tissue (GALT), by direct examination of CD4+ T cells in gut biopsies. However, we did not find a dramatic decrease in these cells in GALT in HIV+ subjects on ART, despite the wide belief that CD4+ T cells are irrevocably lost from GALT during primary HIV-1 infection [48]. In contrast, we previously reported that Tregs are a relatively minor subpopulation of CD4+ T cells in GALT biopsies from HIV-uninfected controls, typically with median 2–7% of CD4+ T cells [40], reflecting low expression of integrin α4β7+ on circulating Tregs. When compared to the medians of the CD73+ subset of CD4+ T cells of 10–15% in the same tissues, it suggests possible limitation of Tregs driving adenosine production by CD73+ CD4+ T cells in this tissue. The combination of trafficking of CD73+ CD4+ T cells through GALT and dependence on IL-7 is consistent with the known important role of IL-7 in homeostasis of lymphocytes in GALT [49].

High levels of CCR5 expression on CD73+ CD4+ T cells suggests susceptibility to HIV-1 entry, and we found similar levels of HIV DNA in CD73+ and CD73-negative memory CD4+ T cells, firstly in untreated HIV+ subjects and then also in patients on fully suppressive ART. Although CD73+ CD4+ T cells had a resting phenotype of very low CD38 and Ki-67 expression and high Bcl-2, suggesting reduced in vivo proliferation, which could mean potentially reduced permissiveness to HIV-1 infection, we have consistently found that resting CD127^high^ CD4+ T cell subsets contain HIV DNA [41,50,51,52].

The vigorous proliferative response of CD73+ CD4+ T cells in vitro may be due not only to their propensity to produce IL-2, consistent with previous results for CD73+ CD8+ T cells [6], but also partly due to the specific separation from CD39+ CD4+ T cells known to be suppressive in vitro [4,7,53,54], or Tregs expressing the checkpoint inhibitor TIGIT [32].

We found that CD73+ CD4+ and CD8+ T cells include effector cells with high expression of TCF-1, which has also been demonstrated in murine quiescent memory T cells that are capable of proliferative responses in response to recall antigens [30]. TCF-1 is integral to Wnt/β-catenin signaling, but whether this signaling pathway is involved in memory T cell differentiation is still unclear [29,55,56]. High TCF-1 expression is also maintained during homeostatic proliferation [29], as we similarly found with maintenance of CD73 expression during IL-7 driven proliferation in vitro.

A substantial fraction of vaccinia-specific CD4+ T cells were also CD73+, even up to 10 years after inoculation, suggesting that CD73+ antigen-specific CD4+ T cells can be extremely long-lived cells. It has previously been reported that vaccinia-specific CD4+ T cells were particularly long-lived, up to 40–60 years [57]. However, HIV-infected subjects tended to lose these responses, compared to healthy controls, and this loss persisted despite otherwise apparent CD4 immune reconstitution [58]. These observations are consistent with a significant fraction of vaccinia-specific CD4+ T cells being contained in the CD73+ subset, which is clearly greatly reduced in chronic HIV-1 infection and not reconstituted in the circulation following ART. Expression of CD127 and responsiveness to IL-7 in vitro, and high levels of Bcl-2, further suggest that longevity of CD73+ antigen-specific CD4+ T cells is mainly due to IL-7 signaling in vivo. The role of IL-7 in human T cell development is well established, and studies in murine models demonstrate an essential role in ongoing T cell homeostasis [59]. Whether recombinant IL-7 therapy in HIV+ subjects that boosts CD4+ T cell numbers [60] also increases CD73+ CD4+ T cells has not been studied.

CD73+ CD4+ T cells were generally not cytotoxic, by phenotype and transcriptome analysis, but we did find CD73+ CD4+ T cells with cytotoxic effector molecules in CMV seropositive healthy adults, and CD73+ CD8+ CTL in all donors. There were relatively higher mRNA levels in CD73+ CD4+ T cells for a distinctive molecule, NKTR, which has been described in NK cell cytotoxicity [61] and also reported in some T cells [62], a finding that requires further study. The existence of proliferative cytotoxic effector CD4+ T cells is consistent with our earlier findings of memory HIV- and CMV-specific CD4+ CTL that proliferated in response to specific antigens in vitro [26].

The preferential depletion of CD73+ CD4+ T cells in HIV-infection may be due to a combination of susceptibility to infection and a general recruitment of resting memory cells into the CD73-negative CD38+ pool. Loss of CD73+ CD4+ T cells may partly explain one of the most typical features of HIV-1 infection, namely reduced in vitro proliferative responses to typical CD4 recall antigens. This is associated with reduced IL-2 production [63], but its cellular basis has never been fully explained in patients with only moderately reduced CD4+ T cell counts. Loss of CD73+ cells specific for Mtb, CMV and HSV may also be important in vivo, since they are important opportunistic infections in advanced HIV-1 infection.

Nevertheless, the most significant finding of the current study is that CD73+ memory CD4+ T cells can be infected, but not completely lost in HIV-infection, and instead, during ART, they could represent a particularly refractory reservoir for latent HIV-1 infection. Long-lived resting CD73+ memory CD4+ T cells dependent on low-level IL-7 signaling for survival, but having a very low level of turnover in vivo compared to the CD73-negative subset, may help maintain an HIV-1 reservoir for years [42].

In summary, CD73+ memory CD4+ T cells mediate proliferative recall responses to important pathogens that represent some of the prevalent opportunistic infections in progressive HIV-1 infection, but at the same time highly express CCR5 and are susceptible to infection, and are not readily reconstituted after ART. It is possible that these cells may be best preserved by early intervention with ART, including CCR5 blocking antiretroviral therapy, such as Maraviroc, commenced during primary HIV-1 infection, although a lack of reconstitution may necessitate intervention, for example with exogenous IL-7, or revaccination.

## 4. Materials and Methods

### 4.1. Subjects

Eight primary HIV-1 infection (PHI) and 8 chronic HIV-1 infection (CHI) subjects were enrolled in the PINT study, as previously described [35] and analyzed prospectively before and after commencement of an antiretroviral (ART) regimen containing an integrase inhibitor. A further 10 PHI subjects were also recruited to the PHIIDO study, a prospective observational study of subjects who did not commence ART [27], and samples from viremic long-term survivors and aviremic elite controllers enrolled in the Australian long-term non-progressor (LTNP) cohort, as previously described [50]. Cryopreserved PBMC samples were also studied from CHI subjects commencing ART in the PRIRIS study [36] and the OZCOMBO study [37] and from untreated CHI subjects in a leukopheresis study [41]. Subjects undergoing vaccinia inoculation were also studied, as previously described [27,39]. Sixteen HIV-uninfected adult control subjects and thirteen HIV-infected subjects (on suppressive ART), undergoing routine endoscopy and colonoscopy screening were recruited to provide gut biopsies, as previously described [40]. Hepatitis C virus (HCV) infected subjects and HCV/HIV coinfected patients, and acute EBV patients, have been previously reported [64,65].

### 4.2. Ethics

Healthy adult controls were recruited from university and hospital staff and students, as approved by the St Vincent’s Hospital Human Research Ethics Committee (HREC/10/SVH/130 (2010)). Enrolment of subjects in the PINT, OZCOMBO, vaccinia, leukopheresis, gut biopsy, HCV and acute EBV studies have been previously described in detail [27,35,37,40,41,64,65]. PHIIDO cohort studies were approved by St Vincent’s Hospital Sydney HREC (HREC/08/SVH/180 (2008)). All subjects gave written informed consent.

### 4.3. Immunophenotyping

The CD73+ subset of memory CD4+ T cells was routinely analyzed in freshly collected ACD or sodium heparin anticoagulated whole blood. Immunophenotyping was performed as previously described [27,28,35]. Monoclonal antibodies used in this study are listed in Supplementary Table S1. Fresh whole blood samples were stained, according to manufacturers’ directions, and lysed with Optilyse C (Beckman Coulter, Indianapolis, IN, USA) for 8- or 12-colour analysis on a four-laser LSRII (BD Biosciences, San Jose, CA, USA) as previously described [27,35], or 18-colour analysis on a 5-laser LSRFortessa X-20, or 20-colour analysis on a FACSymphony (BD Biosciences) as previously described [28]. Intracellular staining was done using transcription factor buffer reagents (BD Biosciences) according to the manufacturer’s directions. Compensation was checked daily and HIV-infected patient samples were run in parallel with healthy adult control samples.

### 4.4. CyTOF Analysis

PBMC were prepared from fresh ACD-anticoagulated blood, within 2 h of venesection, using Ficoll-Paque Plus (GE Healthcare, Chicago, IL, USA) as previously described [27] and 9 × 10^6^ PBMC were stained in a volume of 300 µL with mAb for mass cytometry analysis on a CyTOF 2 (Fluidigm, San Francisco, CA, USA) as previously described [28,66]. The monoclonal antibodies and metal tags used are shown in Supplementary Table S1. In particular, CD73-PE (BD Biosciences) was used for first step labeling of PBMC, and anti-PE-Gd156 (BioLegend) was used as the second step labeling reagent (Supplementary Table S1).

### 4.5. Cell Sorting and Lymphoproliferation Assays

For cell sorting experiments, PBMC were stained with the following mAb conjugates: CD3-PerCP-Cy5.5, CD4-PE-Cy7, CD73-PE (BD Biosciences) and CD45RO-ECD (Beckman Coulter). The CD73+ and CD73-negative subsets of CD45RO+ CD4+ CD3+ T cells were isolated by cell sorting using a FACSAria cell sorter (BD Biosciences) as previously described [27,67]. RNA extraction and quantitative RT-PCR assays were done as previously described [68].

For in vitro proliferation assays, PBMC were labeled with CellTrace Violet (CTV; Thermo Fisher, Waltham, MA, USA), according to the manufacturer’s directions, as previously described [53,67].

Purified subsets of memory CD4+ T cells were resuspended in Iscove’s modified Dulbecco’s medium (IMDM, Life Technologies, ThermoFisher, Waltham, MA, USA) containing glutamine and 10% human AB serum (Sigma-Aldrich, St Louis, MO, USA). These cells were then added to cultures containing autologous adherent cells in 96-well flat-bottom plates (Falcon, Corning, NY, USA). Control cultures consisted of adherent cells alone, and an unstimulated control culture. Purified CD73+ and CD73-negative memory CD4+ T cells from different healthy adults were incubated with *Staphylococcus* enterotoxin B (SEB; 1 µg/mL; Sigma-Aldrich) or recall antigens that had previously been shown to activate CD4+ T cells from those individuals [33], including cytomegalovirus (CMV) lysate (1/500 dilution; Grade III Antigen; Meridian Life Science, Memphis, TN), herpes simplex virus-1 (HSV-1) lysate (1/100 dilution) or *Mycobacterium tuberculosis* (MTb) lysate (1/50 dilution) [33]. Cultures were incubated for 7 days in a humidified 5% CO_2_ incubator at 37 °C, and proliferation was measured as CD25+CTV^dim^ CD4+ T cells as previously described [67].

### 4.6. Antigen-Specific CD4+ T Cell Assays

Intracellular cytokine assays were performed as previously described [14,26]. Briefly 0.5 mL fresh sodium heparin anticoagulated blood was incubated with SEB or antigens (as above) for 2 h at 37 °C, before the addition of 10 µg/mL brefeldin A (Sigma-Aldrich), and incubated for a further 4 h, before addition of 2 mM EDTA. Aliquots were stained with CD3-PerCP-Cy5.5, CD4-PE-Cy7, CD8-APC-Cy7 and CD73-PE, lysed with FACSLyse (BD Biosciences), permeabilized with FACSPerm2 (BD Biosciences), and then incubated with anti-IL-2-APC (BD Biosciences), IFN-γ-Pacific Blue (BioLegend, San Diego, CA, USA) or –FITC (BD Biosciences) and IL-17-AF488 (BioLegend). Cells were then analyzed on an LSR II, as described above.

CD25+CD134+ (“OX40”) assays were performed as previously described [33]. Briefly, 0.25 mL IMDM was added to 0.25 mL fresh Na Hep anticoagulated blood and incubated with SEB or antigens (as above) for 40–48 h at 37 °C. Aliquots were stained with CD3-PerCP-Cy5.5, CD4-PE-Cy7, CD25-APC, CD134-FITC and CD73-PE, lysed with Optilyse C, and then analyzed on an LSR II, as described above.

### 4.7. Ultrasound-Guided Lymph Node Fine Needle Biopsies

Ultrasound-guided lymph node (LN) fine needle biopsies (FNB) were performed as previously described [23], under local anesthesia, using four passes of a 25-gauge needle into a single inguinal LN. Cells were transferred into 10 mL Iscove’s modified Dulbecco’s medium (IMDM; ThermoFisher, Waltham, MA, USA) supplemented with 10% fetal calf serum (FCS; Bovogen, East Keilor, Australia), pelleted by centrifugation at 400 × *g* for 7 min, then resuspended in 1 mL IMDM/FCS for staining, within 1 h of biopsy [23].

### 4.8. Gut Biopsies

Sixteen healthy adult control subjects and thirteen HIV-infected subjects undergoing routine endoscopy and colonoscopy screening provided ten pinch biopsies taken from each of the three sites: left colon (LC), right colon (RC) and terminal ileum (TI). All subjects had no abnormalities detected during the procedure. CD4+ T lymphocytes were studied in detail in single cell suspensions prepared from these biopsies, as previously described [40]. Immunophenotyping for CD73+ CD4+ T cells in gut biopsies was performed and analyzed on the LSR II flow cytometer, as described above for PBMC, except that all tubes contained anti-CD45 and -EpCam mAb for initial gating of lymphocytes and exclusion of epithelial cells [40].

### 4.9. HIV DNA Quantitative PCR

HIV DNA was measured in cell sorted subsets of CD4+ T cells as previously described [35,41,42].

For samples of PBMC from untreated patients, DNA was extracted from CD4+ subsets, purified by cell sorting, using the Qiagen DNeasy Blood and Tissue kit (Hilden, Germany) and quantified by a real-time PCR assay specific for HIV-*gag* with a lower limit of detection (LLOD) of 10 copies. Briefly, 800 nM of the sense primer SK145 5′- AGTGGGGGGACATCAAGCAGCCATGCAAAT-3′ and antisense primer SKCC1B 5′- TACTAGTAGTTCCTGCTATGTCACTTCC-3′ were used in conjunction with 200 nM of the dual-labeled fluorogenic TaqMan locked nucleic acid probe SKLNA2-3 5′-6-FAM AT[C]A[A]T[G]AGGAA[G]CT[G]C-BHQ-1-3′, and iQ Supermix (Bio-Rad Laboratories California, Benicia, CA, USA). PCR conditions consisted of 1 cycle of 95 °C for 3 min followed by 40 cycles of 95 °C for 15 s and 60 °C for 1 min. Total HIV-1 DNA was normalized with genomic DNA, using the Applied Biosystems (Foster City, CA, USA) TaqMan beta-actin detection reagents with a FAM-labeled probe, as previously described [35,41].

For PBMC samples from patients on ART who had fully suppressed plasma viral load <50 copies/mL for at least 2 years, genomic DNA was extracted from isolated cell populations using the direct lysis method [42]. Total HIV DNA levels were quantified by qPCR targeting a fragment within the *pol* gene (primers and probes are listed in Supplementary Table S4), and normalized for total genomic DNA using the TaqMan β–actin detection kit (Life Technologies), as previously described [42]. Reactions contained 25 μL iQ Supermix (Bio-Rad Laboratories), 1 μM mf299 and mf302, 100 nM ri15 and ri16, 10 μL DNA template and dH_2_O to a final volume of 50 μL. Reactions were incubated at 95 °C for 3 min, followed by 45 cycles of 95 °C for 15 s then 60 °C for 1 min. The dynamic range of the total HIV DNA assay as determined by the plasmid (pNL4-3) standard curve (mean ± SD) was 3 × 10^6^ copies (quantitative threshold cycle (Cq) = 17.70 ± 0.47) to 3 copies (Cq = 37.22 ± 1.00) per reaction. Mean (±SD) values for the β- actin positive control were 9.07 ± 1.6 ng/μL [42].

### 4.10. Microarray Analysis

CD73+ and CD73- subsets of memory CD4 T cells and of CD8 T cells were isolated by cell sorting as described above. RNA was purified using an RNeasy Micro Kit (Qiagen, Courtaboeuf Cedex, France) and quantified using a ND-8000 spectrophotometer (NanoDrop Technologies, Fisher Scientific, Illkirch Cedex, France) before being checked for integrity on the 2100 BioAnalyzer (Agilent Technologies, Massy Cedex, France). Labeled cRNA was hybridized on Illumina Human HT-12v4 bead chips following the instructions of the Illumina protocol. Quality controls were checked using Illumina GenomeStudio software. Raw data were filtered both on detection average signal *p*-values (*p* ≤ 0.05) and the proportion per group threshold of 0.5. The lumi bioconductor package algorithm included in the Flexarray software was used to preprocess and normalize Illumina microarray data. The background was corrected using RMA background adjustment followed by log2 to stabilize variance and quantile normalization. Differentially expressed genes were analyzed using local-pooled-error test (LPE) [69]. Genes with a fold change ≥ 1.5 and false discovery rate (FDR) ≤ 0.05 were considered statistically significant. The microarray data have been deposited in the MIAME compliant database Gene Expression Omnibus (http://www.ncbi.nlm.nih.gov/geo/, GEO Series accession number GSE160805).

### 4.11. Statistical Analysis

Results are presented as medians and interquartile ranges. Paired comparisons of the results for CD73+ versus CD73- cells were performed by Wilcoxon signed rank test, using PRISM (v6.0e for MacOSX, GraphPad Software, San Diego, CA, USA). Two tailed *p* values < 0.05 were considered significant.

## Data Availability

The microarray data have been deposited in the MIAME compliant database Gene Expression Omnibus (http://www.ncbi.nlm.nih.gov/geo/, GEO Series accession number GSE160805).

## Supplementary Materials

The following are available online at https://www.mdpi.com/1422-0067/22/2/912/s1.
