# Supplementary material for: CD73+ CD127high Long-Term Memory CD4 T Cells Are Highly Proliferative in Response to Recall Antigens and Are Early Targets in HIV-1 Infection
Source: Int J Mol Sci. 2021 Jan 18;22(2):912. doi: 10.3390/ijms22020912 (PMC7831934; doi:10.3390/ijms22020912)
Supplement: Supplementary file 1 [file ijms-22-00912-s001.zip › ijms-1009852-suppl 2.0/Supplementary Figure Legends.docx]

Supplementary Figure Legends

Supplementary Figure 1

1. Summary data of T cell markers on CD73+ CD45RO+ memory CD4+ T cells
2. Representative flow plots of CD73+ CD4+ T cells showing levels of expression of T cell markers of differentiation, trafficking, cytokine receptor, activation and cytotoxic effector molecules
3. Reduced expression of CCR7 on CD73+ CD45RO+ memory CD4+ T cells, compared to CD73-negative CD45RO+ memory CD4+ T cells and CD45RO-negative naive CD4+ T cells.

Supplementary Figure 2

1. Comparison of 20-colour flow cytometry data for CD73+ CD45RO+ CD4+ T cells versus CD45RO+ Tregs in the same sample shown for one healthy adult control, donor IVPP18.
2. Concordance between high dimensional analysis of FACSymphony fluorescence flow cytometry data and CyTOF mass cytometry for CD73+ CD45RO+ memory CD4+ T cells for healthy adult C005
3. Concordance between high dimensional analysis of FACSymphony fluorescence flow cytometry data and CyTOF mass cytometry for CD73+ CD45RO+ memory CD4+ T cells for healthy adult C001

Supplementary Figure 3 – Representative flow plots showing reduced CD73+ expression and increased CD38 expression on CD45RO+ CD4+ T cells at baseline for a PHI subject from Figure 6A.

Supplementary Figure 4 – Comparison of CD73+ cells as percentage of CD8+ T cells in healthy adult controls, primary HIV-1 infection (PHI) subjects, chronic HIV-1 infection (CHI) subjects, HCV+ subjects, HIV+/HCV+ co-infected subjects and acute EBV subjects.

Supplementary Figure 5 - Prospective longitudinal data of CD73+ CD45RO+ memory cells as percentage of CD4+ T cells in n=13 late-stage CHI subjects commencing antiretroviral therapy (ref [1]), compared to healthy adult control values (left graph). Results are from cryopreserved samples from baseline (BSL) to week 24. Prospective longitudinal data of activated CD38+HLA-DR+ as percentage of CD4+ T for the same patients and controls is shown in the right graph.

Supplementary Figure 6 - Absolute cell counts of CD73+ CD45RO+ memory CD4+ T cells in single cell suspensions prepared from left colon (LC), right colon (RC) or terminal ileum (TI), from subjects that were HIV-uninfected controls (HUC), versus HIV+ subjects on ART (HIV+). CD4 T cell numbers (left graph) and CD8 T cell numbers (right graph) have been normalized to the number of epithelial cells recovered in the single cell suspensions as described in reference [2].

1. Seddiki, N.; Sasson, S. C.; Santner-Nanan, B.; Munier, M.; van Bockel, D.; Ip, S.; Marriott, D.; Pett, S.; Nanan, R.; Cooper, D. A.; Zaunders, J. J.; Kelleher, A. D., Proliferation of weakly suppressive regulatory CD4+ T cells is associated with over-active CD4+ T-cell responses in HIV-positive patients with mycobacterial immune restoration disease. *Eur J Immunol* **2009,** 39, (2), 391-403.

2. Zaunders, J.; Danta, M.; Bailey, M.; Mak, G.; Marks, K.; Seddiki, N.; Xu, Y.; Templeton, D. J.; Cooper, D. A.; Boyd, M. A.; Kelleher, A. D.; Koelsch, K. K., CD4+ T Follicular Helper and IgA+ B Cell Numbers in Gut Biopsies from HIV-Infected Subjects on Antiretroviral Therapy Are Similar to HIV-Uninfected Individuals. *Front Immunol* **2016,** 7, 438.
