# Supplementary material for: CD73+ CD127high Long-Term Memory CD4 T Cells Are Highly Proliferative in Response to Recall Antigens and Are Early Targets in HIV-1 Infection
Source: Int J Mol Sci. 2021 Jan 18;22(2):912. doi: 10.3390/ijms22020912 (PMC7831934; doi:10.3390/ijms22020912)
Supplement: Supplementary file 1 [file ijms-22-00912-s001.zip › ijms-1009852-suppl 2.0/Supplementary Table S1 and S4.docx]

Supplementary Table 1 – Monoclonal antibodies used in this study

| **mAb/**  **reagent** | **FACS-Symphony Clone** | **Fluoro-chrome** | **Supplier** | **CyTOF Clone** | **Metal**  **Tag** | **Supplier*** |
| --- | --- | --- | --- | --- | --- | --- |
| CD73 | AD2 | PE | BD |  |  |  |
| CD73 | AD2 | BUV737 | BD |  |  |  |
| CD73 | AD2 | BV421 | BioLegend |  |  |  |
| CCR5 | REA245 | PE-Vio770 | Miltenyi Biotec | REA245-Biotin | - | Miltenyi Biotec |
| Anti-Biotin |  |  |  | 1D4-C5 | Nd150 | BioLegend |
| CD45 |  |  |  | HI30 | Pd110 | BD |
| CD49d | 9F10 | BV510 | BioLegend | 9F10 | Pr141 | BioLegend |
| CD19 |  |  |  | HB19 | Nd142 | BD |
| CD45RA | HI100 | BUV 563 | BD | HI100 | Nd143 | BioLegend |
| CD4 | L3T4 | BUV496 | BD | RPA-T4 | Nd145 | BioLegend |
| CD8a | SK1 | BUV805 | BD | RPA-T8 | Nd146 | BD |
| CD28 | CD28.2 | BB515 | BD | CD28.2 | Nd148 | BioLegend |
| CLA |  |  |  | HECA-452 | Sm149 | BioLegend |
| CD123 |  |  |  | 6H6 | Eu151 | BioLegend |
| CD161 | 191B8 | Biotin | Miltenyi | DX12 | Gd152 | BD |
| Streptavidin |  | BUV395 | BD |  |  |  |
| CD62L | DREG-56 | APC-eFluor780 | eBioscience | DREG-56 | Eu153 | BioLegend |
| CCR6 | G034E3 | BV421 | BioLegend | REA-190 | Gd154 | Miltenyi |
| Anti-PE |  |  |  | PE001 | Gd156 | BioLegend |
| CCR4 | 205410 | PE | R&D | L291H4 | Gd158 | BioLegend |
| CCR7 |  |  |  | 150503 | Tb159 | R&D |
| CD14 |  |  |  | M5E2 | Gd160 | BD |
| CD45RO |  |  |  | UCHL1 | Dy161 | BioLegend |
| CXCR3 | 1C6/CXCR3 | APC | BD | G025H7 | Dy163 | BioLegend |
| CD49f |  |  |  | GOH3 | Er164 | Fluidigm |
| CD127 | HIL-7R-M21 | BV786 | BD | A019D5 | Ho165 | BioLegend |
| CXCR5 | J252D4 | PE-CF594 | BioLegend | RF8B2 | Er166 | BD |
| CD27 | M-T271 | APC-R700 | BD | M-T271 | Er167 | BioLegend |
| CD25 | M-A251 | PE-Cy5 | BD | M-A251 | Tm169 | BioLegend |
| CD3 | SK7 | PerCP-Cy5.5 | BD | UCHT1 | Er170 | BioLegend |
| CD38 | HIT2 | BUV661 | BD | HIT2 | Yb172 | Fluidigm |
| Integrin ß7 | FIB04 | BV605 | BD | FIB04 | Yb173 | BD |
| HLA-DR | G46-6 | BV711 | BD | L243 | Yb174 | BD |
| CD56 |  |  |  | NCAM16.2 | Lu176 | BD |
| DNA1† |  |  |  |  | Ir191 | Fluidigm |
| DNA2† |  |  |  |  | Ir193 | Fluidigm |
| Granzyme K | GM6C3 | PE | Santa Cruz |  |  |  |
| Anti-Biotin | Bio3-18E7 | VioBright515 | Miltenyi Biotec |  |  |  |
| Granzyme B | GB11 | APC, AF700 | Thermo Fisher |  |  |  |
| Granzyme  A | CB9 | AF488 | BioLegend |  |  |  |
| TIA-1 | 2G9 | PE | Beckman Coulter |  |  |  |
| TCF-1 | C63D9 | AF647 | Cell  Signalling |  |  |  |
| T-bet | O4-46 | PE-CF594 | BD |  |  |  |
| TIGIT | MBSA43 | PE | Thermo  Fisher |  |  |  |
| CD25 | 2A3 | APC | BD |  |  |  |
| CD134 | L106 | PE | BD |  |  |  |

* Clones were conjugated with metal tags by Ramaciotti Facility for Human Systems Biology, Sydney University, except for conjugates supplied by Fluidigm, as indicated.

†Intercalators

Supplementary Table 4 – Primers and probes for HIV *pol* qPCR

| **Primer/ probe** | **Sequence (5’ → 3’)** | **5’ mod** | **3’ mod** | **bp** | **Binding site*** |
| --- | --- | --- | --- | --- | --- |
| mf299 | GCA CTT TAA ATT TTC CCA TTA GTC CTA |  |  | 27 | 2536‐ 2562 |
| ri15 | CAG [G]A[A] T[G]G [A]TG G | 6‐ FAM | BHQ‐ 1 | 13 | 2590‐ 2602 |
| ri16 | CTG [T]C[A] A[T]G [G]CC A | 6‐ FAM | BHQ‐ 1 | 13 | 2619‐ 2631 |
| mf302 | CAA ATT TCT ACT AAT GCT TTT ATT TTT TC |  |  | 29 | 2634‐ 2662 |

Brackets indicate locked nucleic acids. Mod = modification; 6‐FAM = 6‐Carboxyfluorescein; BHQ‐1 = Black Hole Quencher 1. *Relative to HIV B reference genome (HXB2). qPCR = quantitative PCR.
