# Supplementary figures and images for: CD73+ CD127high Long-Term Memory CD4 T Cells Are Highly Proliferative in Response to Recall Antigens and Are Early Targets in HIV-1 Infection
Source: Int J Mol Sci. 2021 Jan 18;22(2):912. doi: 10.3390/ijms22020912 (PMC7831934; doi:10.3390/ijms22020912)

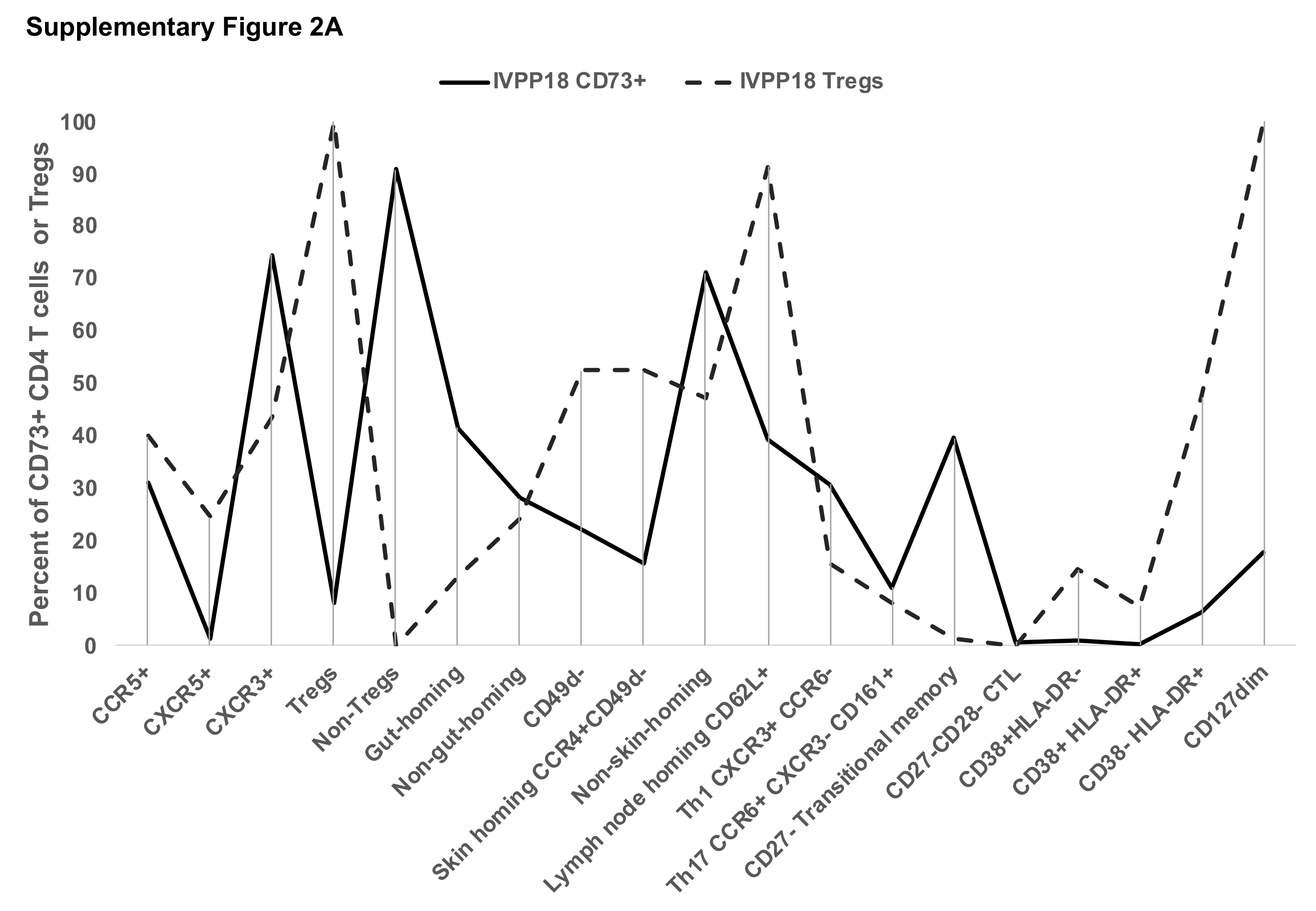

Supplement: Supplementary file 1 [file ijms-22-00912-s001.zip › ijms-1009852-suppl 2.0/Supplementary 2A.tif]

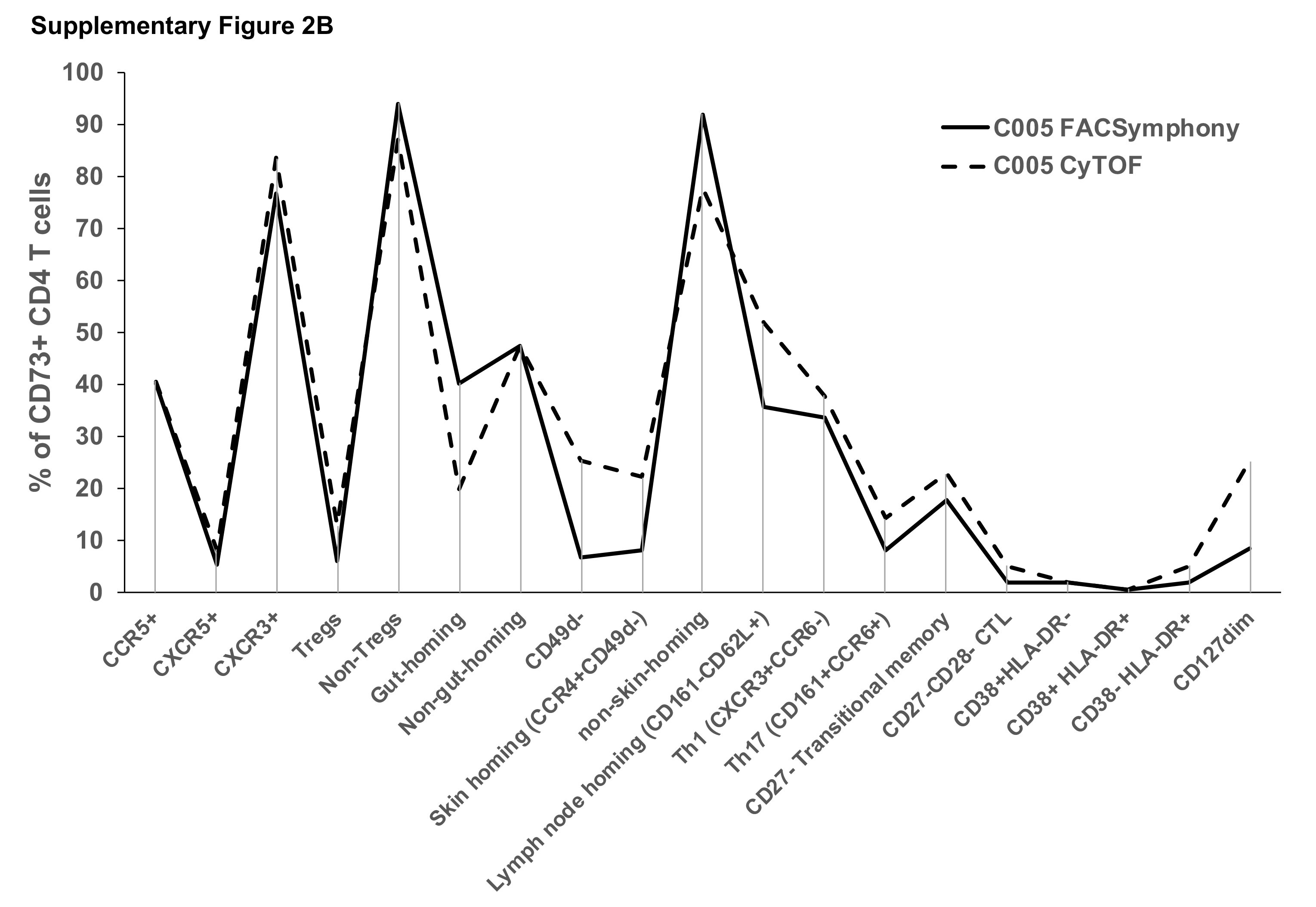

Supplement: Supplementary file 1 [file ijms-22-00912-s001.zip › ijms-1009852-suppl 2.0/Supplementary 2B.tif]

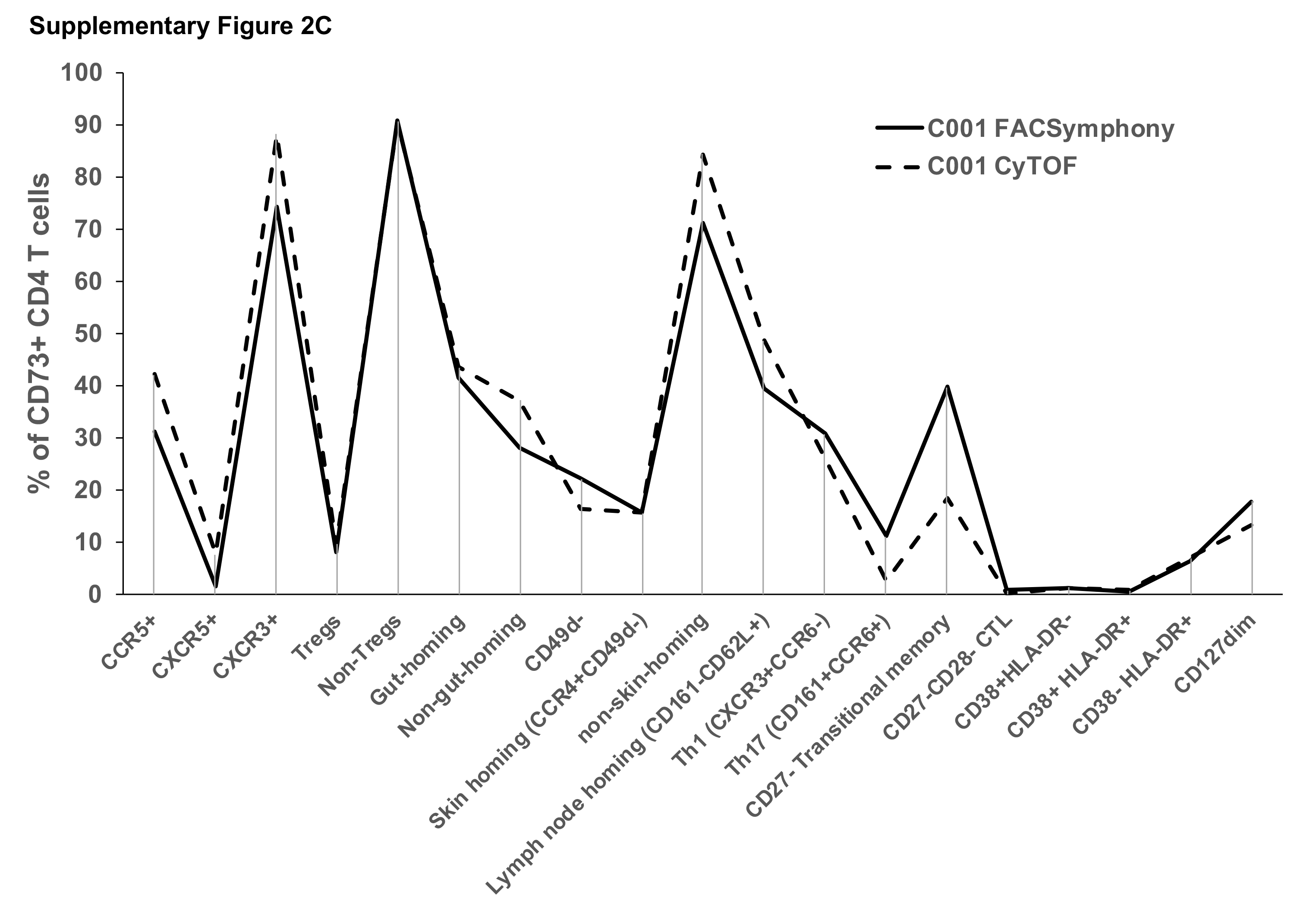

Supplement: Supplementary file 1 [file ijms-22-00912-s001.zip › ijms-1009852-suppl 2.0/Supplementary 2C.tif]

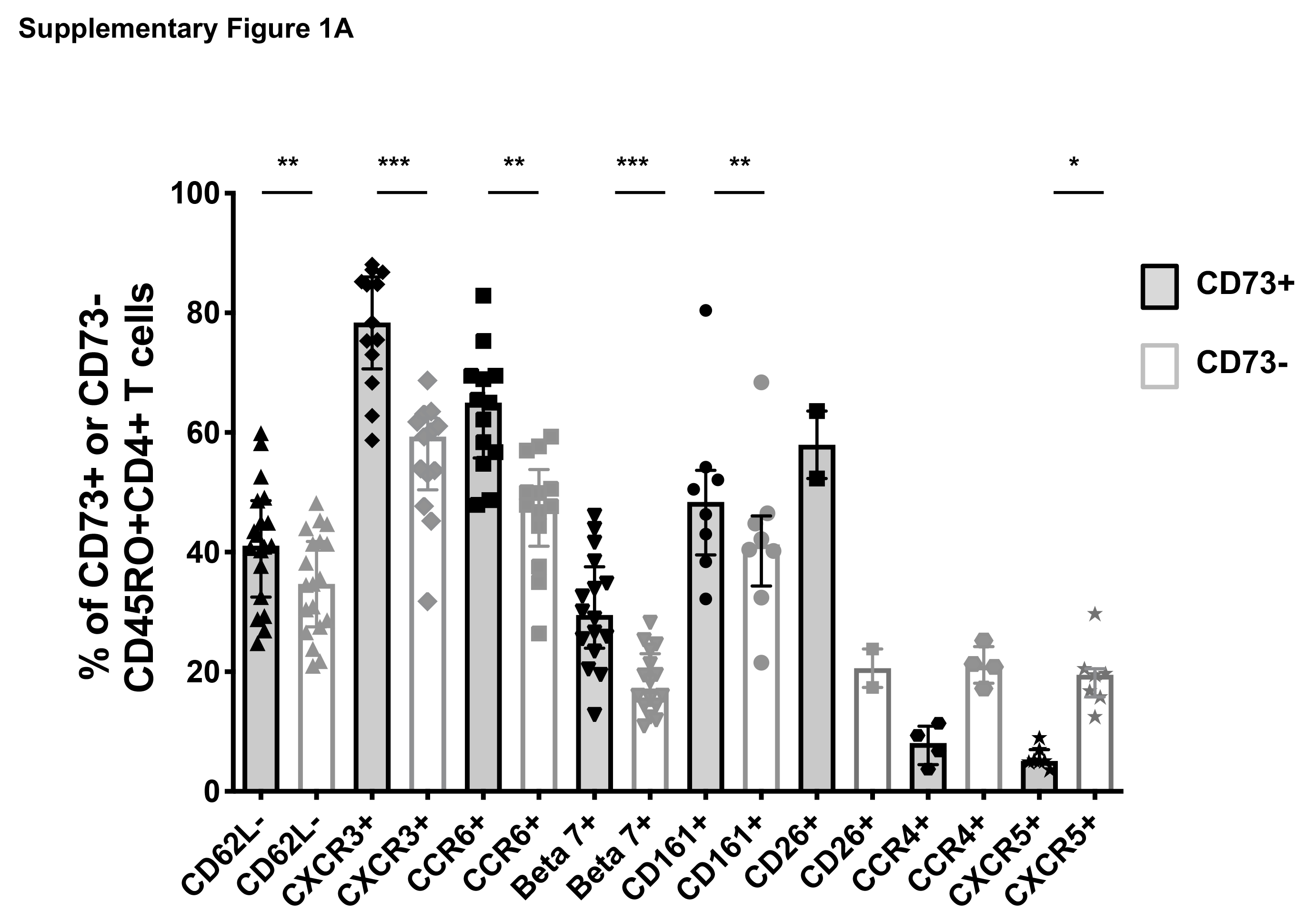

Supplement: Supplementary file 1 [file ijms-22-00912-s001.zip › ijms-1009852-suppl 2.0/Supplementary Figure 1A.tif]

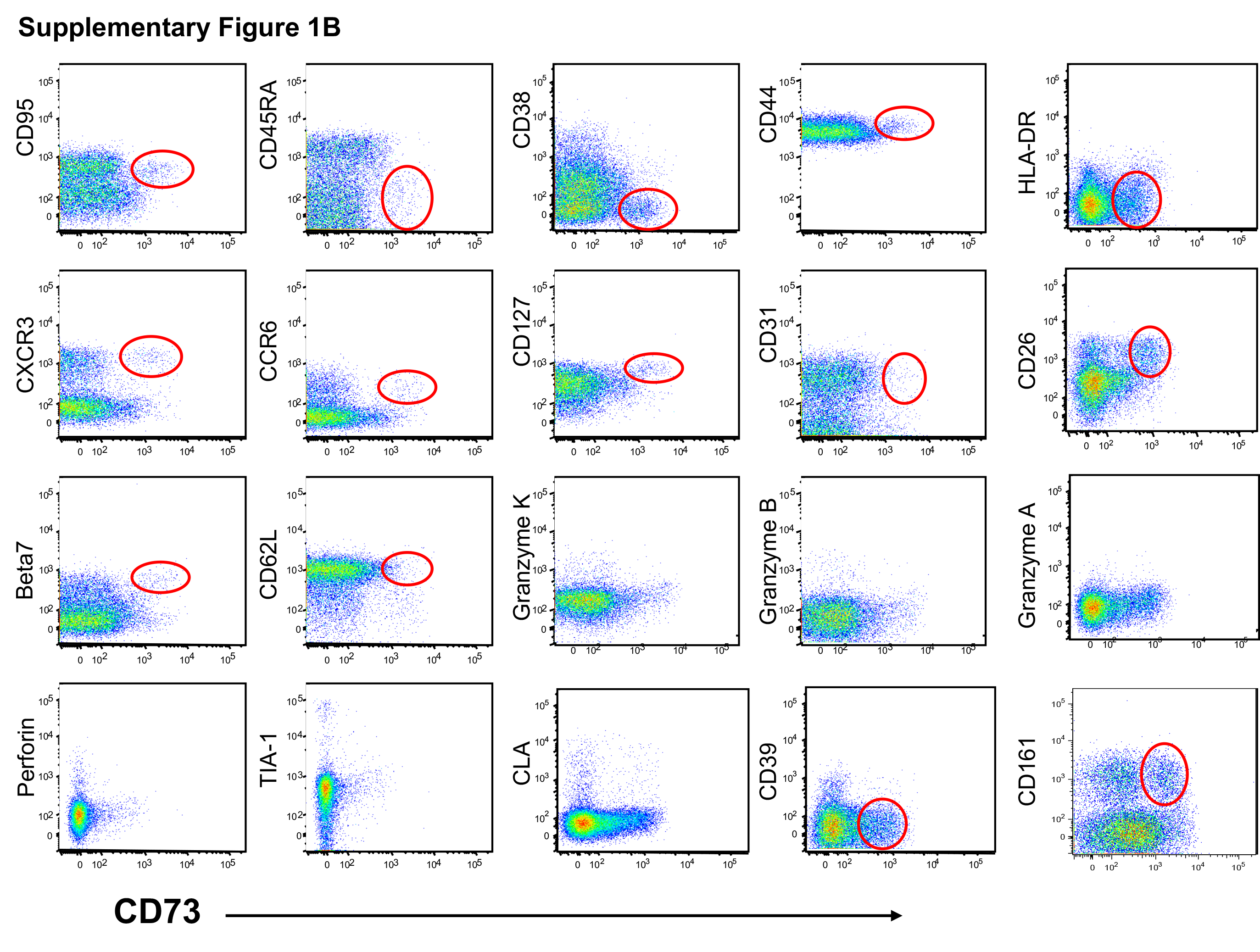

Supplement: Supplementary file 1 [file ijms-22-00912-s001.zip › ijms-1009852-suppl 2.0/Supplementary Figure 1B.tif]

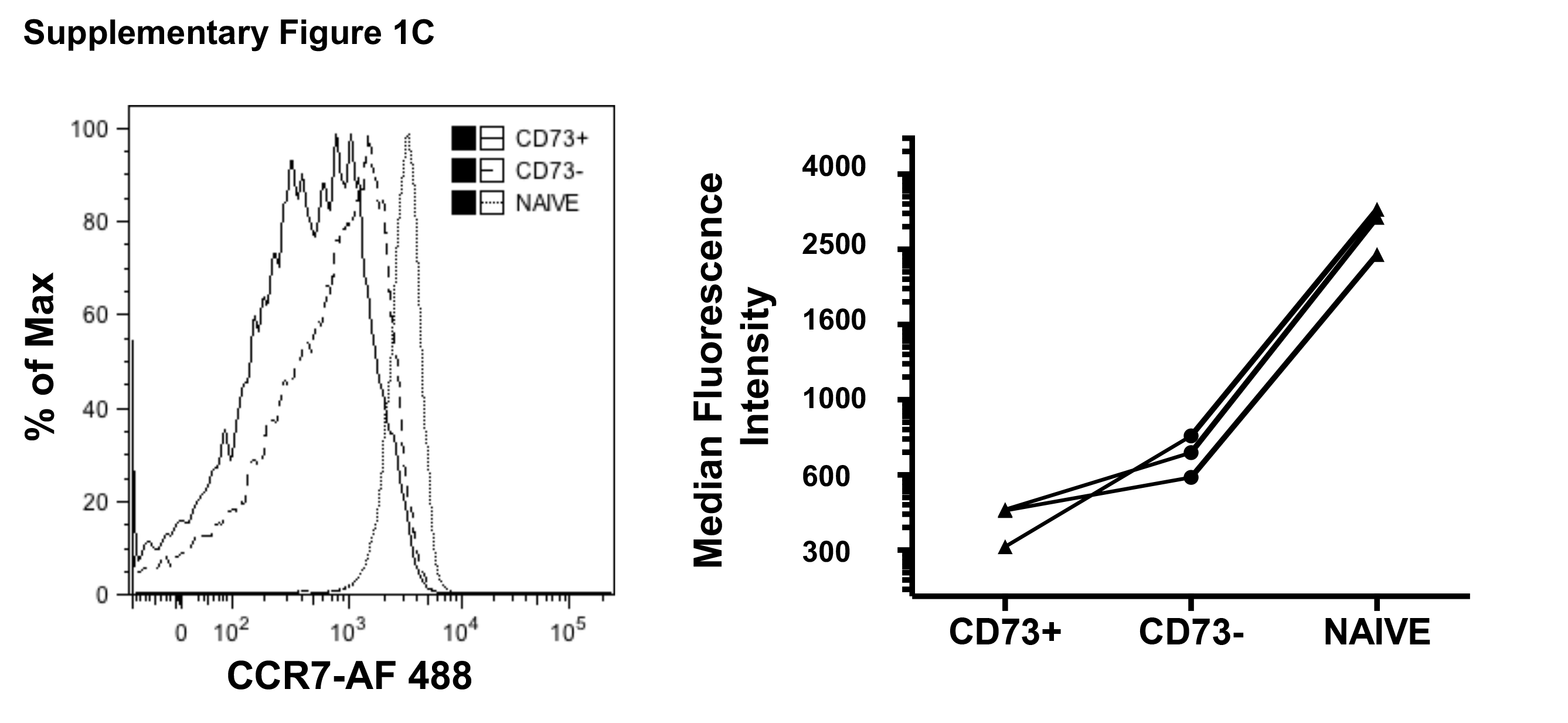

Supplement: Supplementary file 1 [file ijms-22-00912-s001.zip › ijms-1009852-suppl 2.0/Supplementary Figure 1C.tif]

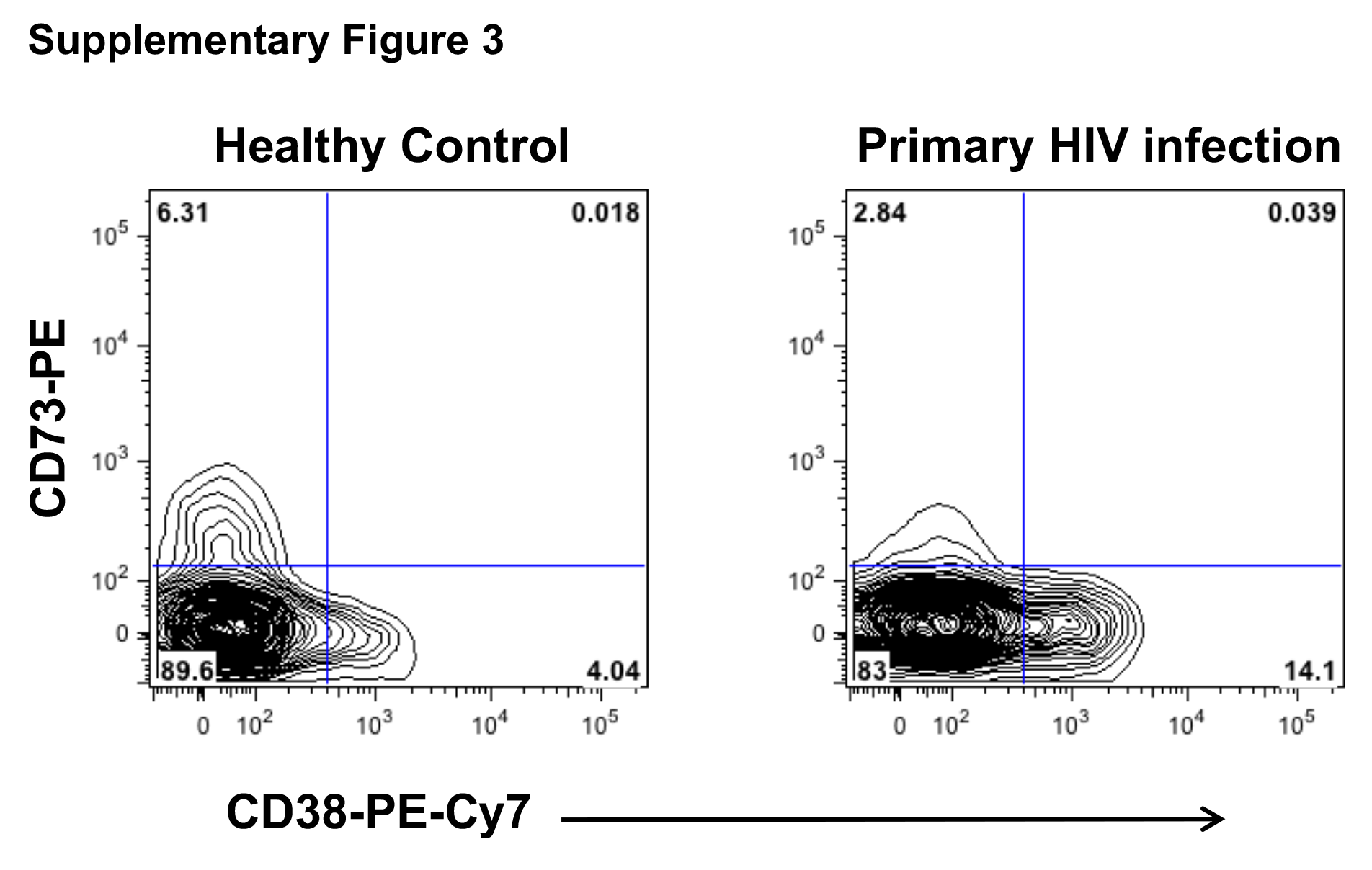

Supplement: Supplementary file 1 [file ijms-22-00912-s001.zip › ijms-1009852-suppl 2.0/Supplementary Figure 3.tif]

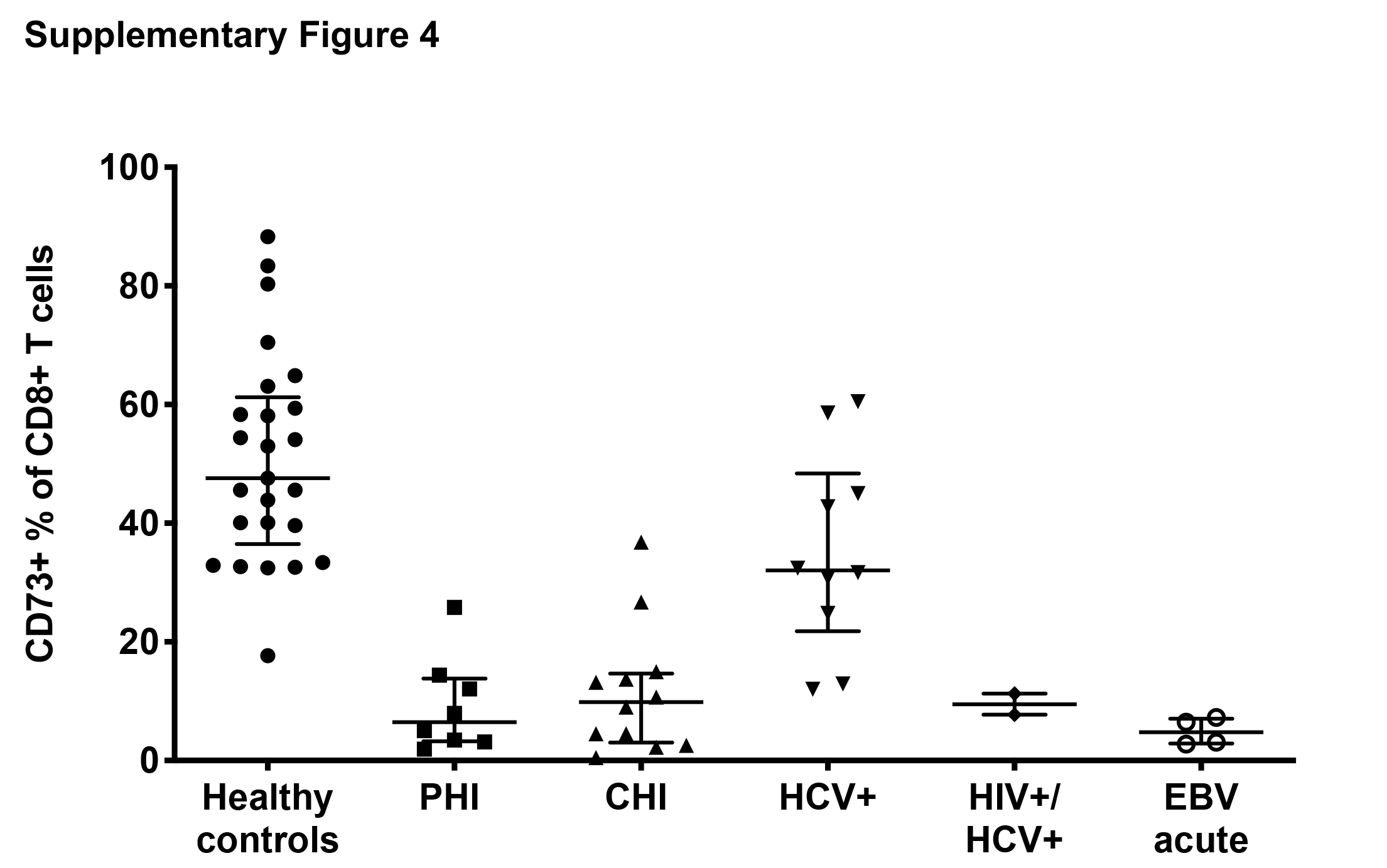

Supplement: Supplementary file 1 [file ijms-22-00912-s001.zip › ijms-1009852-suppl 2.0/Supplementary Figure 4.tif]

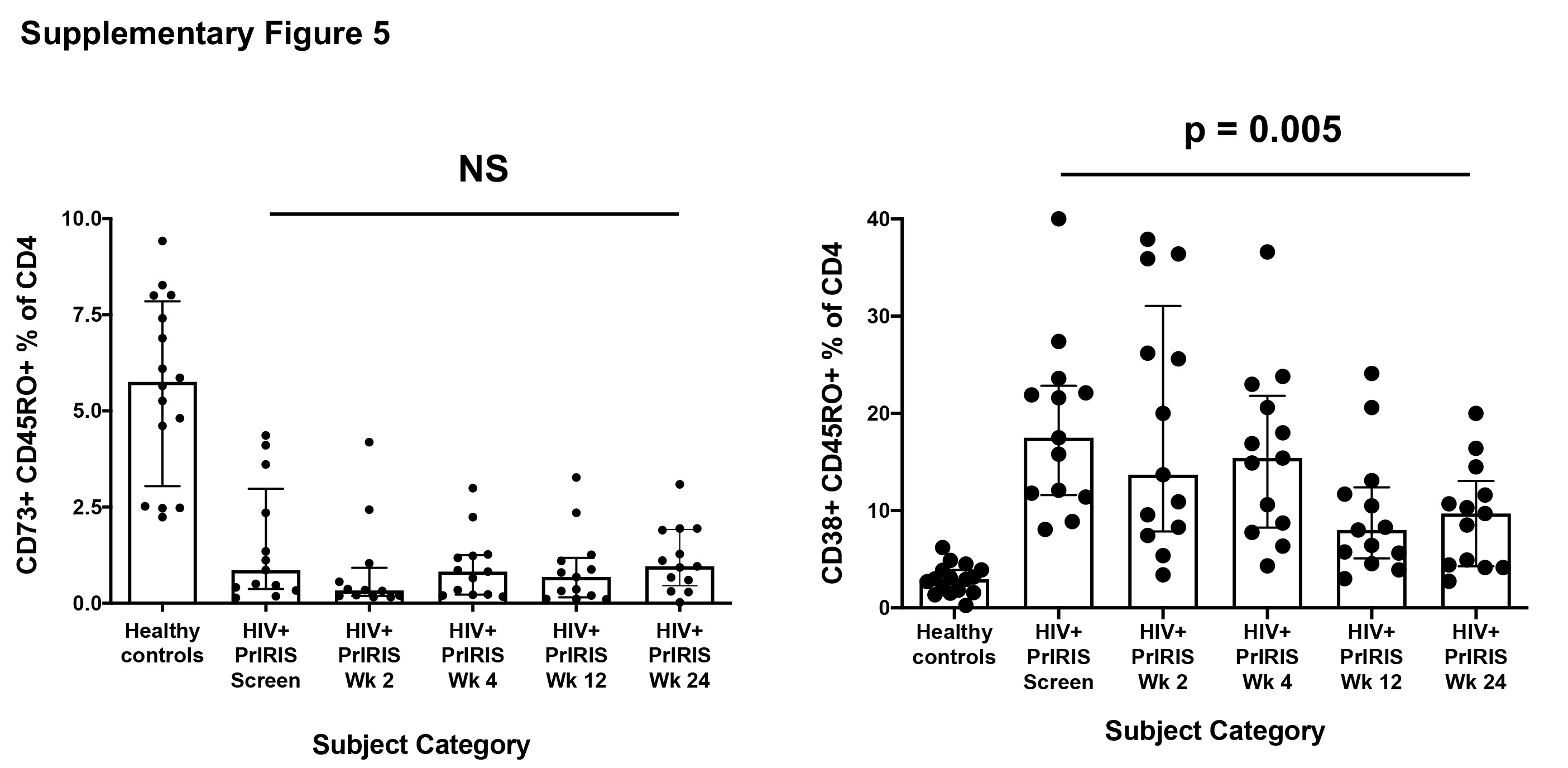

Supplement: Supplementary file 1 [file ijms-22-00912-s001.zip › ijms-1009852-suppl 2.0/Supplementary Figure 5.tif]

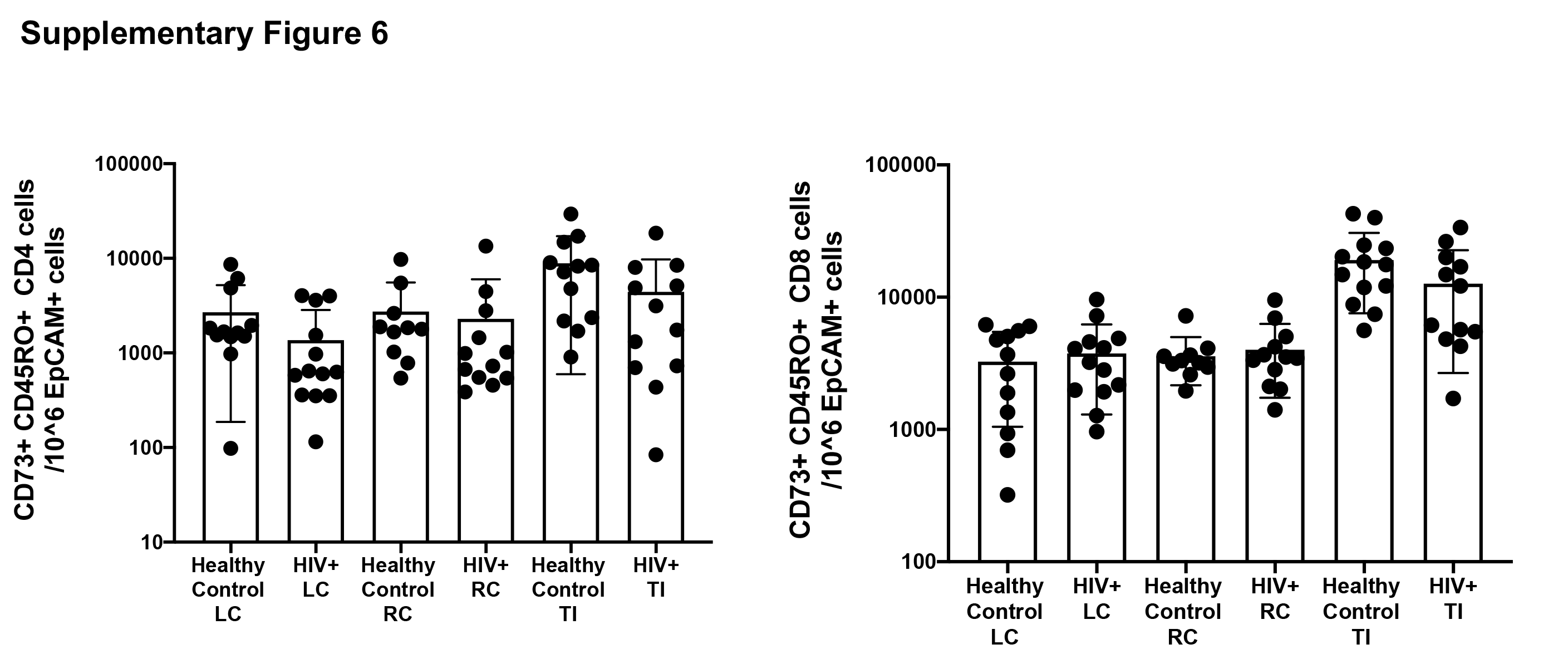

Supplement: Supplementary file 1 [file ijms-22-00912-s001.zip › ijms-1009852-suppl 2.0/Supplementary Figure 6.tif]
